# Are Semantic Representations Stable? A Bayesian Framework Applied to the Study of Quantifier Meaning

**DOI:** 10.1007/s42113-026-00302-x

**Published:** 2026-04-27

**Authors:** Alexandra Sarafoglou, Anne S.F. Giacobello, Henrik R. Godmann, Tamar Johnson, Ingmar Visser, Julia M. Haaf, Jakub Szymanik

**Affiliations:** 1 Department of Psychology, University of Amsterdam, Amsterdam, The Netherlands; 2 Department of Psychological Research Methods, University of Potsdam, Potsdam, Germany; 3 Center for Brain/Mind Sciences and the Department of Information Engineering and Computer Science, University of Trento, Trento, Italy

**Keywords:** Semantic representations, Individual differences, Bayes factors, Inequality constraints

## Abstract

Researchers have begun using Bayesian hierarchical modeling to study semantic representations, for instance, in the context of natural language quantifiers such as *most*, *few*, and *more than half*. Building on previous work, we propose a Bayesian hierarchical model to disentangle three key semantic parameters: the meaning threshold of quantifiers, the vagueness surrounding meaning thresholds, and response noise. We use this model to test the stability of semantic representations over time and across different paradigms. To examine stability over time, we analyzed existing data ($n=63$) from Ramotowska et al. (2023). Contrary to the conclusions drawn by the original authors, we found overwhelming evidence in favor of the hypothesis that semantic representations change over time ($\text {BF} > 10^{304}$). At the same time, we found overwhelming evidence that the relative ordering of meaning thresholds within individuals remained stable ($\text {BF} = 4 \times 10^{24}$). Next, we conducted a new experiment ($n=178$) to test stability across paradigms, specifically comparing a linguistic paradigm to a visual one. Here too, we found overwhelming support for differences in between-subject variability in meaning thresholds across paradigms ($\text {BF} = 7.48 \times 10^{30}$) and for differences in vagueness ($\text {BF} = 1.17 \times 10^{110}$). Our findings challenge the assumption that semantic representations of logical vocabulary have stable, fixed values, while suggesting that their relative ordering remains stable within individuals. The model we propose provides an effective framework for studying the semantics of quantifiers, detecting individual-level effects, and explicitly accounting for potential instability.

## Introduction

A longstanding area of research in semantics focuses on how individuals assign meaning to words and expressions. The theory in this research area is highly formalized and rich, and usually embedded in logical formalism. For instance, the meaning of an expression is often understood as knowing the conditions under which it is true (Frege, 1903; Wittgenstein, 1921). These truth conditions, also referred to as meaning thresholds, are then formalized mathematically so that the word’s meaning can be expressed unambiguously (e.g., Heim & Kratzer, 1998; Coppock & Champollion, 2019). This way of thinking about meaning relies on several assumptions. One assumption is that each quantifier has a single, well-defined meaning that can be formally expressed. Another assumption is that meaning is stable–that it is an inherent property of a word or expression, shared across individuals. Additionally, it assumes that meaning remains stable within an individual; if it can be separated from pragmatic influences, it should be consistent over time and across different modalities (e.g., using the same adjective to describe both a written object and a picture of that object).

While logical semantics provides precise theoretical predictions, its definitions of meaning often do not align with individuals’ response behavior in experimental studies. For instance, individuals may interpret words and expressions differently, or their behavior may challenge the assumption that meaning is stable. This has led researchers to enrich their logical theories with probabilistic information, for instance, through fuzzy logic (Zadeh, 1983) or by describing individuals’ response behavior through statistical models with semantically meaningful parameters (e.g., van Tiel et al., 2021; Ramotowska et al., 2024), which we refer to as *semantic representations*. Similarly, this work examines meaning by operationalizing it as a semantic representation, with a particular focus on the stability of these representations. Specifically, we ask whether semantic representations, as captured by the parameters of our computational model, remain stable across time and experimental paradigms.

In the following sections, we present empirical findings essential for studying variability in semantic representations in experimental settings and introduce an extension of the Bayesian hierarchical model proposed by Ramotowska et al. (2024) informed by these findings. The proposed statistical model is specifically designed to analyze empirical data from sentence-verification paradigms and focuses on the semantic representations of single-bounded quantifier words, that is, words that express quantity and have only one meaning threshold (e.g., *more than half*) rather than two (e.g., *some, but not all*; *about half*; see Szymanik (2016) for an overview). However, our statistical model and the methodologies discussed here are also applicable to the broader study of meaning.

**Fig. 1:**
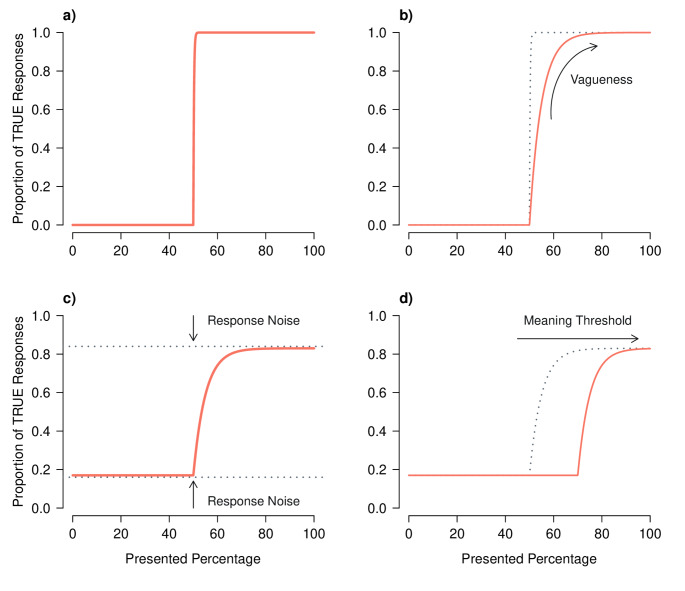
Hypothetical response behavior of an individual for the quantifier *more than half* under the proposed model. The individual exhibits (**a**) an optimal response performance (top row, left); (**b**) a response pattern with uncertainty around the meaning threshold (top row, right); (**c**) a response pattern with uncertainty around the threshold and response noise (bottom row, left); (**d**) a response pattern with a shifted threshold, uncertainty around the threshold, and response noise (bottom row, right)

Crucially, the proposed model aligns with the tradition of threshold models and characterizes response behavior in terms of meaning thresholds, vagueness, and response noise. Figure 1 presents a schematic illustration of an individual’s response behavior for the quantifier *more than half* under the model in a sentence-verification paradigm. Specifically, it shows the probability of judging a quantifier statement as TRUE as a function of different presented percentages assuming (a) optimal response performance of the individual, (b) a response pattern including vagueness, (c) one including both vagueness and response noise, and (d) one including vagueness, response noise, and a shifted meaning threshold.

### Logical Theories of Meaning

Since its establishment, Generalized Quantifier Theory (Barwise & Cooper, 1981; Keenan & Stavi, 1986; Szymanik, 2016; Mostowski, 1979; Peters & Westerståhl, 2006) has emerged as the main theoretical foundation for representing and validating quantifiers in experimental semantics. Its strength lies in its capacity to provide a framework for formalizing quantified statements such as “Most cats are pets”. Within this theory, common determiners such as *few*, *many*, or *most* are treated as relations between sets of individuals (i.e., “cats” and “pets”), which allows their truth conditions to be expressed through set theory. Put formally, the phrase “*Most* cats are pets” is considered true if the number of elements in the intersection between “cats” and “pets” (i.e., $|(cats \cap pets)|$) is greater than the difference between “cats” and “pets” (i.e., $|(cats - pets)|$). This formal approach allows for an unambiguous definition for each quantifier if the threshold is clearly defined. An example where this is not the case is “*Many* cats are pets” in which the truth-conditional value depends on a threshold that needs to be established based on its uses.

When researchers apply predictions from Generalized Quantifier Theory to empirical data, they often attribute deviations from their logical definition as pragmatic effects or response noise (e.g., Kotek et al., 2015; Ariel, 2004; Hackl, 2009; Pietroski et al., 2009; Lidz et al., 2011).^1^ However, even in experiments using pseudowords that lack real-world meaning and contextual associations, it remains challenging to explain the wealth of empirical data using Generalized Quantifier Theory alone. Specifically, we find that individuals’ response behavior differentiates quantifiers along three key dimensions: the meaning threshold, the vagueness surrounding meaning thresholds, and response noise.

### Variation in Meaning Threshold, Vagueness, and Response Noise

The dimensions that likely receive the most attention in the experimental semantics literature are the meaning threshold and vagueness, particularly when testing empirically whether truth-conditionally equivalent quantifiers are uttered for similar proportions. Consider the two quantifiers *most* and *more than half*. Despite being commonly considered to be truth-conditionally equivalent, corpus studies (Solt, 2016, 2011), experimental studies (Ariel, 2003; Denic & Szymanik, 2022; Kotek et al., 2015; Talmina et al., 2017; Ramotowska et al., 2023), and modeling studies (Carcassi et al., 2021), but see Hackl (2009) indicate that the quantifier *more than half* has its meaning threshold near 50%, whereas *most* is associated with higher proportions.

The differentiation between vague and sharp meaning thresholds was discussed by Kennedy (2007) and rests on three key characteristics. First, expressions with vague meaning thresholds exhibit contextual variability, whereby individuals assign different truth conditions in different contexts. For instance, the quantifier *many* may refer to different quantities when speaking of “*Many* bananas in a fruit bowl” or “*Many* people on the New York City subway”. Second, vague quantifiers are susceptible to borderline cases. Individuals often struggle to make definitive judgments when asked to verify the truth of an expression that is near their individual meaning threshold. That is, an individual evaluating the statement “*Many* people on the New York City subway” may consider 1,000 to be many, 200 not many, but be unsure when determining the truth value of 600 people.^2^ Finally, vague quantifiers lead to the Sorites Paradox, which arises when removing a single unit from the whole does not invalidate the truth of the sentence (e.g., if 600 people on the New York City subway are considered *many*, then 599 people are also considered *many*), thereby suggesting that this process could be repeated until only one unit remains (i.e., one person). In linguistic tasks, Ramotowska et al. (2023); Denic and Szymanik (2022) found that individuals struggle with borderline cases for the quantifier *most*, but not for *more than half*, as they exhibit longer response times and greater inconsistencies when verifying sentences near the meaning thresholds.

Examining individuals’ response noise reveals additional systematic differences. Response noise refers to the extent to which an individual deviates from their optimal response, for instance, due to mistakes or lapses in attention. Well-recognized in the literature is the polarity effect, which shows that response noise is lower for upward-entailing quantifiers compared to downward-entailing quantifiers when their thresholds are sharp (i.e., *more than half*, *fewer than half*; Wason (1961); Just and Carpenter (1971); Clark (1976); Grodzinsky et al. (2018); Reißner et al. (2024)), but also when they are vague (i.e., *many*, *few*; Deschamps et al. (2015); Grodzinsky et al. (2018)).^3^ Moreover, when accounting for perceptual difficulties in visual stimuli using Weber’s law, response noise was shown to be independent of the proportion against which the quantifier was validated (Deschamps et al., 2015). This suggests that response noise and vagueness are separate processes, as (1) response noise occurs across all proportions against which the quantifier is validated, while vagueness is restricted to the meaning threshold, and (2) a polarity effect is observed for quantifiers with sharp meaning thresholds and those with vague ones. Recognizing the importance of this distinction, Denic and Szymanik (2022) and Ramotowska et al. (2024) explicitly included response error, further denoted as response noise, into their statistical models to examine monotonicity effects and disentangle the impact of response noise from meaning thresholds and vagueness. Using all three parameters in their statistical model, Ramotowska et al. (2024) confirmed differences in vagueness between quantifiers (i.e., higher values for vague than for sharp-meaning quantifiers), differences in response noise (i.e., higher values for downward-entailing than upward-entailing quantifiers), and substantial individual variation in meaning thresholds.

### Variation Across and Within Individuals

Besides differences between quantifiers across meaning threshold, vagueness, and response noise, we also find meaningful differences between individuals. For instance, studies conducted by Denic & Szymanik (2022) and Ramotowska et al. (2023) revealed that meaning thresholds across individuals differ more for the quantifier *most* than *more than half*. Similarly, Yildirim et al. (2016) identified individual differences in the interpretation of *many* and *some*. Importantly, this observed variability cannot be attributed solely to response noise. On the contrary, these differences appear to be systematic, suggesting that they can be captured by probabilistic models Newstead (1988); van Tiel (2014); Wallsten et al. (1986).

Variability between and within subjects has been observed not only in quantifiers but in numerical cognition more generally (e.g., Halberda et al., 2008) where magnitude judgments follow Weber’s law. Moreover, variability is documented in gradable adjectives (e.g., Solt, 2015) where vagueness and response noise were found to be distinct processes that vary both across and within individuals. Similar variability has been found in vagueness related to category formation (Gruenenfelder, 2019; Verheyen et al., 2019), as well as in similarity judgments and feature attributions for common nouns (Martı et al., 2023), where individuals showed reliable and clustered response patterns while still exhibiting systematic individual differences.

The frequent occurrence of between- and within-subject variability aligns with the growing body of literature which emphasizes the importance of accounting for individual differences in language models more generally (e.g., Kidd et al., 2018). In summary, to effectively bridge the gap between theory and behavior, we propose to formalize Generalized Quantifier Theory as a statistical model, that assumes that even for expressions with identical truth-conditional values, genuine differences in semantic representation may exist both between individuals (e.g., varying meaning thresholds for the same quantifier) and within an individual (e.g., differing degrees of vagueness across quantifiers; Solt, 2011; Denic and Szymanik, 2022).

### Stability of Meaning Representation

Finally, to understand the semantic representation of quantifiers, it is important not only to study them at one specific point in time but also to evaluate their stability. The key question is whether individuals settle on a consistent meaning threshold or degree of vagueness, or whether these values fluctuate over time and are better represented as distributions rather than fixed points. These insights can inform theoretical approaches that explore the relationship between cognition and logical theories of meaning, as well as guide research on behavioral interventions for individuals with selective language deficits. However, the evidence on the stability of semantic representations over time remains inconsistent. For instance, in the domain of categorization, Verheyen et al. (2019) found that semantic representations change over time, whereas (Ramotowska et al., 2023), in the domain of quantifier meaning, found evidence of stability over a two-week period. Relatedly, studying the relationships between semantic representations across different paradigms can offer valuable insights, particularly in revealing differences between visual and numerical processing. One possible explanation for these differences is the difficulty of estimating quantities in visual stimuli (Halberda et al., 2008; Dehaene, 1997), which can lead to different response patterns. For instance, previous studies have shown that truth-conditional behavior for *most* and *more than half* is indistinguishable in visual paradigms (Hackl, 2009; Pietroski et al., 2009), but not in linguistic paradigms (Ariel, 2003; Denic & Szymanik, 2022; Ramotowska et al., 2023).

### The Current Paper

Here we apply the proposed computational model in two empirical studies to study differences among quantifiers and to test how stable semantic representations are across paradigms and over time. First, we applied our model to data published by Ramotowska et al. (2023) to study the stability of semantic representation over time. The data for this study features sixty-three individuals who performed a sentence-verification paradigm over two time points. Second, to study whether vagueness and between-subject variability was stable across tasks, we conducted a new experiment. In this experiment 178 individuals performed a linguistic task in which participants verified quantifiers based on sentences, and a visual task, in which they verified quantifiers based on dot patterns. The following section we describe our statistical model and its capabilities in more detail. Section ‘Study 1: Stability of Meaning Across Time’ presents the results of our reanalysis of the Ramotowska et al. (2023) data, section ‘Study 2: Stability Across Paradigms’, presents the results of the new experiment. The paper concludes with a critical reflection on the computational model, potential future extensions, and a discussion of future research questions that could be addressed with this model.

## Methods

### Model Specification

**Fig. 2:**
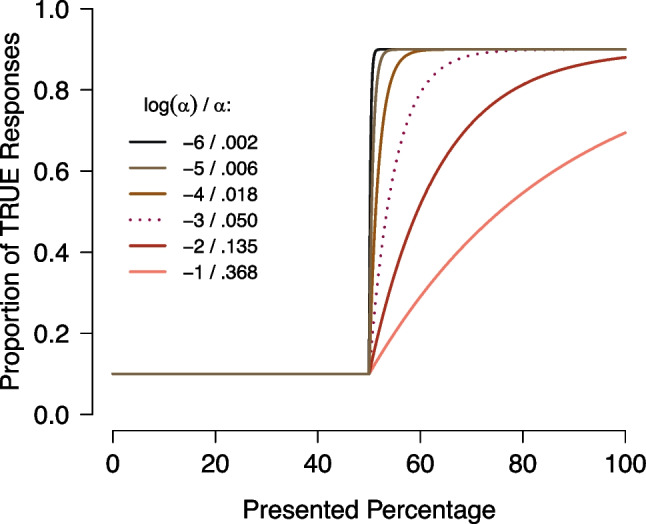
Predicted response behavior for the quantifier *more than half* under the model for a hypothetical individual with varying levels of vagueness around the meaning threshold. Smaller values of $\log (\alpha )$ and $\alpha$ result in sharper meaning thresholds. The dashed line represents the predicted response behavior based on the value favored by the prior distribution assigned in the current study

The structure of the model is based on the Bayesian hierarchical three-parameter logistic regression model proposed by Ramotowska et al. (2024) with three key improvements. First, we aligned the model with semantic intuition, by assigning informative prior distributions that restrict parameters to admissible ranges and generate meaningful predictions. Second, we extended upon its existing structure by adding a covariance structure on the threshold and response noise parameters which allows researchers to assess the relationship between semantic representations across different experimental paradigms and time points. Third, the current model introduces an alternative approach to linking individuals’ response behavior to presented percentages, which hinges on a reinterpretation of the meaning threshold parameter. Probabilistic models on quantifier meaning, including Ramotowska et al.’s model, often use a logistic link, and thus define the meaning threshold parameter as the point at which the probability of considering a quantifier statement TRUE is 50%. However, this approach conflicts with the traditional interpretation of meaning, which is to know the conditions under which an expression is true. In contrast, the current model employs an exponential link that defines the meaning threshold as the necessary and sufficient condition for the truth of the quantifier statement. For instance, in Fig. 2 (black line), when vagueness is minimal, the meaning threshold is the same under both interpretations at a presented percentage of 50%. As vagueness increases, the necessary and sufficient condition for the truth of the quantifier statement remains at 50%, as the response probability only begins to rise beyond that point. In contrast, the point at which the quantifier statement is considered TRUE reaches 0.5 shifts upward with increasing vagueness and reaches approximately 80% at the highest vagueness level shown. Hence, our computational model states that a quantifier statement is considered TRUE if and only if the presented percentage exceeds the meaning threshold. As a result, inconsistent responses below the meaning threshold are attributed only to response noise; inconsistent responses above the threshold are attributed to both vagueness and response noise. Notably, the two link functions can hardly be distinguished in terms of their predictions and thus the primary difference lies in parameter interpretation; the exponential link aligns more closely with semantic theory.

While the previous version of the model predicted a large range of data patterns, our current model has been improved to predict response patterns consistent with semantic theory. To achieve this, we first imposed constraints on the meaning threshold and response noise parameters to eliminate impermissible or implausible values. Specifically, the meaning threshold parameter was constrained to remove thresholds that fall outside the acceptable range (e.g., meaning thresholds smaller than 0 or larger than 100). The response noise parameter was constrained so individuals’ true response noise could be at chance level but no worse. This is because when the response noise parameter exceeds chance level, it leads to a flipped response pattern. For instance, for the quantifier *more than half* individuals would be more inclined to consider percentages below 50% as instances of the quantifier, while percentages above 50% are less likely to be categorized as such. Such behavior contradicts semantic intuition and undermines the monotonicity assumption, which posits that higher proportions should correspond to a higher likelihood of being categorized as instances of a quantifier. For the paradigms used in the present study and the current sample (i.e., healthy adults), response behavior that is worse than chance is indicative of a failure to adhere to task instructions (which warrants the exclusion of the individual) rather than of a meaningful cognitive process of interest. A second major improvement was to impose a hierarchical structure on the meaning threshold and response noise parameters to accommodate similarities across individuals. Finally, we replaced the logistic link function with an exponential link. The refined computational model takes the following form:

1 $$\begin{aligned} Y_{i,j,k,t} \sim \text {Bernoulli}(\pi _{i,j,k,t}). \end{aligned}$$

In this model, $Y_{i,j,k,t}$ is the observed and discrete response for individual *i* judging quantifier *j* in trial *k* at time point/paradigm *t*. It takes on the values $Y_{i,j,k,t} = 0$ if the response is FALSE and $Y_{i,j,k,t} = 1$ if the response is TRUE. The responses depend on the latent response probabilities $\pi _{i,j,k,t}$. The response probabilities are monotonically increasing and modeled using an exponential link $F(\mu _{i,j,k,t}) = 1 - \text {exp}(-\mu _{i,j,k,t})$ for $\mu _{i,j,k,t} > 0$, where $\mu _{i,j,k,t}$ captures how strongly the stimulus supports a TRUE response, relative to the meaning threshold and adjusted for vagueness, specifically:

2 $$\begin{aligned} \mu _{i, j, k, t} = \frac{c_{i,j,k,t} - \beta _{i,j,t}}{\alpha _{i,j,t}}. \end{aligned}$$

The model then adds the response noise parameter, $\gamma _{i,j,t}$, which sets the lower and upper bounds of an individual’s response consistency:

3 $$\begin{aligned} \pi _{i,j,k,t} = \gamma _{i,j,t} + (1 - 2 \gamma _{i,j,t}) \times \text {F}(\mu _{i,j,k,t}). \end{aligned}$$

In the absence of response noise, the response probability changes depending on the presented percentage on a given trial $c_{i,j,k,t}$ as well as the location parameter $\beta _{i,j,t}$ (i.e., the meaning threshold) and the scale parameter $\alpha _{i,j,t}$ (i.e., the vagueness), using the following function:

4 $$\begin{aligned} \text {F}(\mu _{i,j,k,t}) = {\left\{ \begin{array}{ll} 1 - \text {exp}\left( - \mu _{i, j, k, t}\right) & \text { if } c_{i,j,k,t} \ge \beta _{i,j,t} \\ 0 & \text { if } c_{i,j,k,t} < \beta _{i,j,t}. \end{array}\right. } \end{aligned}$$

In this formulation, for presented percentages above the meaning threshold, the function $\text {F}(\mu _{i,j,k,t})$ transforms $\mu _{i,j,k,t}$ into a probability, such that higher values of $\mu _{i,j,k,t}$ yield higher response probabilities. If the presented proportion is below the meaning threshold, the response probability is set to zero (see Fig. 1, top row). When response noise is introduced, for instance, with a value of $\gamma _{i,j,t} = 0.17$, the curve flattens: the minimum response probability is no longer 0 but 0.17, and the maximum is reduced to 0.83 (i.e., $1 - 0.17$; see Fig. 1, bottom row, left). In the extreme case where response noise reaches $\gamma _{i,j,t} = 0.5$, the individual is effectively responding at chance level: the probability of a TRUE response is 0.5 for all presented proportions, regardless of whether they fall below or above the threshold. This case represents the flattest possible response function, in which the presented proportion has no influence on the response.

The location parameter $\beta _{i,j,t}$ determines the lower bound of the semantic representation for a specific quantifier, its meaning threshold. Note that the presented percentages have been rescaled to proportions and centered at zero, such that a presented percentage of 50% translates to $c_{i,j,k,t} = 0$. The threshold parameters likewise are represented on the same scale; for instance, the thresholds $\beta _{i,j,t} = 0$, $\beta _{i,j,t} = -0.2$, and $\beta _{i,j,t} = 0.3$, indicate that for a given quantifier an individuals’ threshold lies at 50%, 30%, and 80%, respectively. The two top rows in Fig. 1 depict response patterns for individuals who adopt a meaning threshold of 50% (top row left and right, and bottom row left) and 70% (bottom row, right).

The scale parameter $\alpha _{i,j,t}$ determines the steepness of the distribution thus characterizing the vagueness of the threshold. When $\alpha _{i,j,t}$ is zero, indicating no vagueness, the distribution of the response probability takes on a step-shaped form (see Fig. 1, top row, left). In this case, the response probability remains equal to the upper performance bound for all presented proportions above the meaning threshold, while it equals the lower performance bound for proportions below the meaning threshold. However, as the vagueness parameter increases, the distribution gradually flattens, indicating more uncertainty above the threshold (see Fig. 2). Figure 1 (upper row, right), shows the response probability for an individual with a vagueness parameter of $-3$ on the log scale, or 0.05 on its natural scale.

#### Individual-level Parameters

The individual-specific threshold $\beta _{i,j,t}$ and response noise $\gamma _{i,j,t}$ for each quantifier, at one of two specific time points or in one of two specific paradigms, are assumed to be drawn from truncated multivariate normal distributions. The means $(\mu _{j,1}, \mu _{j,2})$ of the multivariate normal distribution represent the expected values of the corresponding parameter at each time point/paradigm. The group-level standard deviations $(\sigma _{j,1}, \sigma _{j,2})$ capture the heterogeneity among individuals, and the correlation coefficient $\rho _j$ relates the two time points/paradigms to each other. Both the threshold parameter and the response noise parameter are truncated within theoretically meaningful boundaries. The permissible range for the response noise parameter ranges between zero (representing optimal response performance) and 0.5 (indicating guessing-level performance), while the threshold parameter is limited to a permissible range between $-0.5$ and 0.5 (corresponding to meaning thresholds between $0\%$ and $100\%$):

5 $$\begin{aligned} \gamma _{i,j,t}&\sim \text {MVNormal} \left( \begin{pmatrix} \mu _{\gamma , j,1}\\ \mu _{\gamma , j,2} \end{pmatrix}, \begin{pmatrix} \sigma _{\gamma , j,1}^2 & \rho _{\gamma , j} \sigma _{\gamma , j,1} \sigma _{\gamma , j,2} \\ \rho _{\gamma , j} \sigma _{\gamma , j,1} \sigma _{\gamma , j,2} & \sigma _{\gamma , j,2}^2 \end{pmatrix} \right) \mathbb {I} (0, 0.5),\end{aligned}$$

6 $$\begin{aligned} \beta _{i,j,t}&\sim \text {MVNormal} \Bigg( \begin{pmatrix} \mu _{\beta , j,1}\\ \mu _{\beta , j,2} \end{pmatrix},\begin{pmatrix} \sigma _{\beta , j,1}^2 & \rho _{\beta , j} \sigma _{\beta , j,1} \sigma _{\beta , j,2} \\ \rho _{\beta , j} \sigma _{\beta , j,1} \sigma _{\beta , j,2} & \sigma _{\beta , j,2}^2 \end{pmatrix} \Bigg)\mathbb {I} (-0.5, 0.5). \end{aligned}$$

The log-vagueness parameter $\text {log}(\alpha )$ for both time points/paradigms is assumed to be drawn from two independent normal distributions. In our model, we deliberately chose not to impose a covariance structure on this parameter. This decision was informed by simulation studies conducted during the model development phase. These simulations revealed that we were unable to recover potential correlations between vagueness parameters across different time points/paradigms. We assigned prior distributions to the logarithm of the vagueness parameter rather than directly modeling $\alpha$, as this allowed us to more easily assign a prior distribution that favors values close to zero and better reflects the theoretical assumption that sharp-meaning quantifiers are associated with precise thresholds and minimal vagueness:

7 $$\begin{aligned} \text {log}(\alpha _{i,j,t})&\sim \text {Normal}(\mu _{\alpha , j,t}, \sigma _{\alpha , j,t}^2) \end{aligned}$$

#### Group-level Parameters

The group-level means for the response noise parameter are drawn from a truncated normal distribution centered at 17%. We centered the distribution towards the middle of its permissible range, rather than near the extremes (which would favor either very low or very high response noise), due to the uncertainty we imposed on both the between-subjects heterogeneity and the correlation parameter. This adjustment was necessary to address potential sampling issues: sampling group-level means near the boundaries of the parameter space, combined with sampling larger values for heterogeneity across individuals, could result in non-permissible values being sampled on the individual level.

8 $$\begin{aligned} \mu _{\gamma , j,t}&\sim \text {Normal}(0.17, 0.05) \mathbb {I} (0, 0.5) \end{aligned}$$

The group-level means of the threshold parameters, too, were assumed to follow a truncated normal distribution. For the quantifiers *fewer than half* and *more than half*, the group-level means ($\mu _{\beta , 1,t}$ and $\mu _{\beta , 2,t}$) were centered on zero with a standard deviation of 0.1. The chosen prior distribution reflects the theoretical assumption that the group-level threshold will be close to or exactly at 50%. The standard deviation ensures that values near the center are mildly favored, while still giving alternative values sufficient weight and avoiding too much prior mass near the boundaries of the permissible range at 0% and 100%:

9 $$\begin{aligned} (\mu _{\beta , 1,t}, \mu _{\beta , 2,t})&\sim \text {Normal}(0, 0.1) \mathbb {I} (-0.5, 0.5). \end{aligned}$$

The prior distributions for the remaining threshold parameters mildly favor values below the 50% mark, specifically, at 30% for the downward-entailing quantifier *few* ($\mu _{\beta , 3,t}$) and values above the 50% mark, specifically, at 70% for upward-entailing quantifiers *many* and *most* ($\mu _{\beta , 4,t}$ and $\mu _{\beta , 5,t}$). Note that the current specification of the model assumes a monotonically increasing response probability as the presented proportion increases and consequently vagueness above the meaning threshold. Downward-entailing quantifiers (e.g., *few*, *fewer than half*) exhibit the opposite pattern in the raw data, that is, higher proportions correspond to lower probabilities of a TRUE response. We therefore reverse the response coding for downward-entailing quantifiers prior to model fitting so that all quantifiers follow a monotonically increasing response function. After model fitting, parameter estimates are transformed back so that they are expressed in terms consistent with the original semantic interpretation of the quantifiers, thus the proportion below which the quantifier is judged to be true. Since the transformation standardizes the direction of the response function (i.e., a monotonically increasing pattern), the prior distributions for these quantifiers are specified identically:

10 $$\begin{aligned} (\mu _{\beta , 3,t}, \mu _{\beta , 4,t}, \mu _{\beta , 5,t})&\sim \text {Normal}(0.2, 0.1) \mathbb {I} (-0.5, 0.5) \end{aligned}$$

The prior distribution on the log-vagueness parameter, $\text {log}(\alpha )$, is centered at $-3$, favoring vagueness values that lie near the middle of the theoretically plausible range:

11 $$\begin{aligned} \mu _{\alpha , j,t}&\sim \text {Normal}(-3, 1). \end{aligned}$$

The standard deviations are drawn from inverse-gamma distributions. The assigned inverse-gamma distributions allow for moderate heterogeneity among individuals for the response noise and threshold parameters and are scaled according to their permissible range (i.e., a broader parameter space allows for greater heterogeneity among individuals):

12 $$\begin{aligned} \sigma _{\gamma ,j,t}&\sim \text {Inv-Gamma}(12, 1) \end{aligned}$$

13 $$\begin{aligned} \sigma _{\beta ,j,t}&\sim \text {Inv-Gamma}(6, 1) \end{aligned}$$

14 $$\begin{aligned} \sigma _{\text {log}(\alpha ),j,t}&\sim \text {Inv-Gamma}(2, 1). \end{aligned}$$

The distribution for the correlation coefficients $\rho$ for the threshold and response noise parameters are obtained by stretching and shifting values from a beta distribution so that they cover the range $[-1, 1]$. The beta distributions are relatively uninformed and symmetric around zero, mildly favoring small to moderate correlations:

15 $$\begin{aligned} r_{\beta ,j}&\sim \text {Beta}(4, 4) \end{aligned}$$

16 $$\begin{aligned} \rho _{\beta ,j}&= (r_{\beta ,j} \times 2) - 1 \end{aligned}$$

17 $$\begin{aligned} r_{\gamma ,j}&\sim \text {Beta}(4, 4) \end{aligned}$$

18 $$\begin{aligned} \rho _{\gamma ,j}&= (r_{\gamma ,j} \times 2) - 1. \end{aligned}$$

#### Model Assumptions

The model is based on several key assumptions. First, the model applies only to single bounded quantifiers, which refer to quantifiers with one threshold (e.g., *more than half*) rather than two thresholds or more (e.g., *about half*; *some, but not all*). Second, the model assumes that individuals perform no worse than chance to ensure that the model respects quantifier monotonicity. Third, it assumes that individuals are drawn from a common distribution, which allows for the capture of individual variation while maintaining a shared underlying structure. Fourth, we assume that response noise affects responses both below and above the meaning threshold, as it reflects random errors rather than systematic bias. This is consistent with the constant error assumption, which states that random errors occur with equal likelihood regardless of whether the proportion is above or below the threshold (see e.g., Ramotowska et al., 2024; Denic and Szymanik, 2022). Finally, the model assumes independence across quantifiers, meaning that the parameters of one quantifier, such as thresholds, vagueness, and response noise, do not impact the parameters of another quantifier. Some literature presents evidence that challenges the independence assumption, which suggests that this assumption may not be entirely realistic. Specifically, findings indicate that individuals do not represent quantifiers in isolation but instead along a mental scale, where the interpretation of one quantifier may shift depending on its position relative to others on this scale (e.g., Ramotowska et al., 2024; Hackl, 2009; Carcassi et al., 2021; Heim et al., 2020). Other literature suggests that cognitive mechanisms for validating quantifier statements may differ across quantifiers, which would necessitate independent modeling (e.g., Denic & Szymanik, 2022; Solt, 2016; Reißner et al., 2024). Due to the lack of consensus on how quantifiers affect each other and whether the impact is universal or limited to quantifiers with certain properties (e.g., vague meaning thresholds), we modeled them independently. Suggestions for expanding the model to account for potential dependencies are provided in the discussion.

#### Model Validation

We validated the proposed computational model to assess whether the prior distributions are consistent with semantic theory and to ensure that the model’s predictions and inferences are reliable. In particular, we evaluated the appropriateness of the prior distributions assigned to the model parameters by visualizing and assessing prior predictive distributions, that is, the distribution of the model parameters and data patterns predicted by the priors (Schad et al., 2021; Gabry et al., 2019; Wagenmakers et al., 2021). The assigned priors should reflect expectations about the parameters and make sensible predictions, which is particularly important for Bayes factor hypothesis testing. In addition to examining prior predictive distributions, we ensured that the proposed computational model, as well as the implemented Markov chain Monte Carlo (MCMC) algorithm were able to (1) recover the prior distribution when no data are observed; (2) that the data effectively update the prior distributions; (3) accurately recover true parameter values. These additional checks were proposed by Kucharsk et al. (2021), based on the recommendations by Talts et al. (2018) and Schad et al. (2021) (see Godmann et al. (2025) for a tutorial paper on model validation methods, specifically tailored to experimental semantics). We conducted these checks using simulated data from 20 participants and 200 experimental trials per participant, suggesting good model performance. The full results can be accessed in the online supplements.

## Study 1: Stability of Meaning Across Time

In study 1, we analyzed data from experiment 2 in Ramotowska et al. (2023). In their study, the authors instructed individuals to judge the truth of a quantifier by completing a sentence-verification paradigm. In their study, the authors’ primary focus was to investigate individual differences and stability of semantic representations of the quantifiers *more than half* and *most*. They found that although the two quantifiers have equivalent truth-conditional meanings, their meaning thresholds were not equivalent. Ramotowska et al. (2023) also reported differences in the steepness parameter of their cognitive model. That is, within their modeling framework, evidence accumulated equally quickly across presented proportions for *more than half*, but slowed down around the meaning threshold for *most*. They interpreted this pattern as reflecting greater uncertainty, or vagueness, around the meaning threshold. Crucially, and most relevant for the present work, Ramotowska et al. (2023) concluded that the meaning thresholds of quantifiers are stable over time. In this reanalysis, we aimed to verify this conclusion. This led to the research question “Are semantic representations stable over time?” from which we derived the following hypotheses:

- $\mathcal {H}_{1,1}$: A statistical model that assumes dependencies among model parameters across time points (i.e., through a covariance structure) outperforms an alternative model that assumes model parameters are stable over time.
- $\mathcal {H}_{1,2}$: If we find evidence in favor of $\mathcal {H}_{1,1}$, we will test whether the ordering of the meaning thresholds remains stable over time for each individual. The alternative model allows all parameters to vary freely.

### Method

#### Participants

Eighty-nine individuals were recruited via Amazon Mechanical Turk, and seventy-two individuals completed both sessions. After exclusion, sixty-three individuals were included in the analysis. The openly available data featured responses from $n = 64$ individuals who were not excluded based on the following criteria: (1) failure to respect quantifier monotonicity ($n = 4$), (2) non-native English speakers ($n = 1$), (3) prior participation in a similar experiment ($n = 0$), and (4) reaction times faster than 300 ms for 50% or more of their responses ($n = 3$). In addition to the aforementioned exclusion criteria, we eliminated responses with peculiar patterns, that is, response patterns which suggest random key presses and non-compliance with task instructions (e.g., individuals pressed only one button for all stimuli, or all response proportions were at chance level). To prevent any exploitation of researchers’ degrees of freedom, the authors determined peculiar patterns through visual inspection of the blinded data (e.g., MacCoun & Perlmutter, 2015; Dutilh et al., 2019), that is, data where the quantifier and session could not be identified and quantifier labels were shuffled across individuals. Two of the authors determined whether the data of any of the participants should be excluded, or, in less severe cases, whether some quantifier conditions should be excluded. Based on this evaluation, 1 out of 64 individuals was excluded, which corresponds to $1.56\%$ of the sample. From the remaining sample, for two individuals, we removed one quantifier condition from the data.

After removing peculiar cases from the dataset, we determined the cutoff criteria for fast and slow responses to exclude trials where participants did not pay attention (slow) or provided an accidental quick button press (fast). Based on the visual inspection of the response time data, we excluded trials in which individuals reacted faster than 200ms or slower than 5000ms for each quantifier and for each answer. That is, from all 31400 trials, we excluded 24 fast responses and 278 slow responses from the data, which corresponds to $0.08\%$ and $0.89\%$ of all trials, respectively.^4^

#### Design

**Fig. 3:**
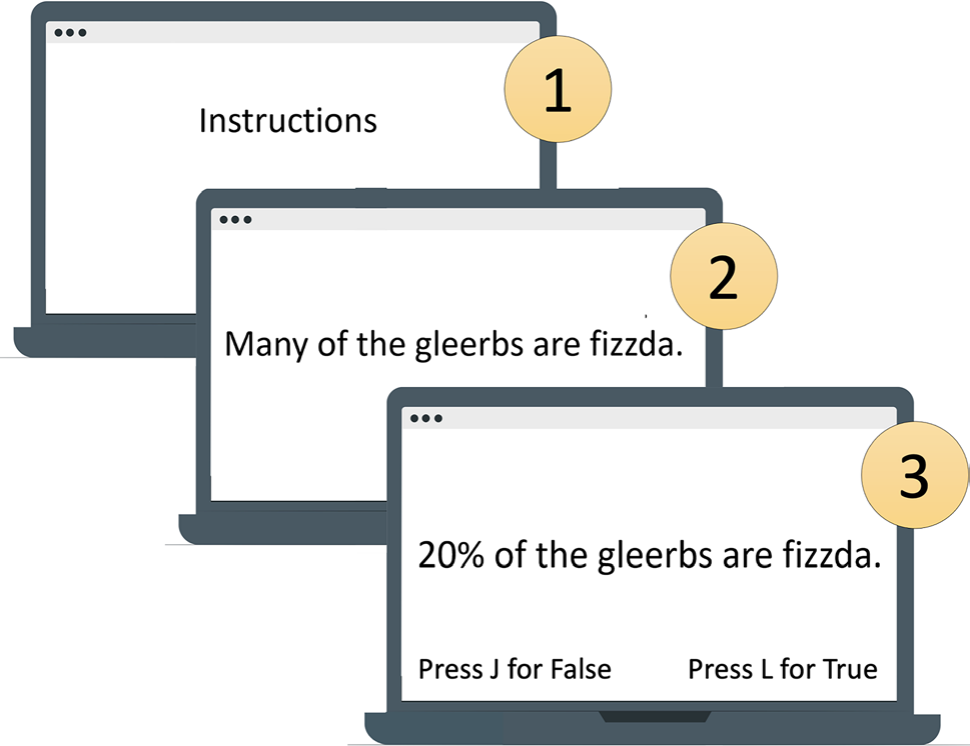
Schematic illustration of the experimental paradigm in study 1. Individuals were asked to first read a quantifier statement, then read a statement containing a percentage, and finally judge whether the statements matched. To minimize pragmatic effects, the statements in study 1 contained pseudowords. Individuals completed this task twice, approximately two weeks apart

In the sentence-verification paradigm, individuals are instructed to judge the truth of a statement containing a quantifier against a given percentage. This task was employed in the following way. Individuals first read a quantifier statement including a pseudo-noun and a pseudo-adjective (e.g., “*Most*/ *Many*/ *Few*/ *More than half*/ *Fewer than half* of the gleerbs are fizzda.”), then read a statement containing a percentage (e.g., “20% of the gleerbs are fizzda.”), and finally judge whether they believed the quantifier statement was TRUE or FALSE based on the percentage statement. The experiment was self-paced, meaning that the statements remained on the screen until the individual pressed a button on the keyboard. Then, the statement containing the percentage appeared, and individuals could indicate their choice. A schematic illustration of the experimental procedure is displayed in Fig. 3.

#### Stimuli

The presented words in the quantifier statements and percentage statements were constructed as follows. A set of 50 pseudo-nouns and 50 pseudo-adjectives were drawn using the software package *Wuggy* (Keuleers & Brysbaert, 2010). These words were then inserted into the quantifier statements, such that each statement featured one pseudo-noun and one pseudo-adjective. For instance, for the quantifier *many*, a quantifier statement could use the pseudo-noun *gleerbs* and the pseudo-adjective *fizzda* to form the statement “Many of the gleerbs are fizzda.”. The statements containing the percentages, which were presented to the individuals afterward, were generated by randomly drawing percentages from a uniform distribution ranging from 1% to 99%, excluding 50%, for each quantifier. For instance, with the randomly drawn percentage of 20%, the statement could be: “20% of the gleerbs are fizzda”.

#### Procedure

Individuals were familiarized with the sentence-verification paradigm by first completing a practice block consisting of 8 trials. The experimental block consisted of 250 trials (50 trials per quantifier) in randomized order. The experimental stimuli were kept the same for all individuals. At the end of the experiment, individuals provided basic demographic information. The same experiment was conducted twice approximately 2 weeks apart.

#### Data Analysis

To test the stability hypothesis, the original authors fitted their statistical model independently in the two sessions and then tested whether the meaning threshold parameters were the same or different across sessions. Our analytic strategy, by contrast, first fits our refined statistical model to the data. The model directly incorporates the potential relationship between sessions. To test the stability hypothesis, we compare the predictive adequacy of this model with one that assumes both sessions can be best described using the same parameters; meaning that the cognitive parameters are the same across sessions. We believe this analytical strategy provides a more comprehensive assessment of the stability of semantic representation since it (1) models a covariance structure between sessions rather than estimating parameters independently, and (2) considers all cognitive parameters of semantic representation, rather than focusing solely on the meaning threshold. To test whether the meaning thresholds retain their relative ordering within each individual, we compared the predictive adequacy of two models. The first assumes a fixed, hypothesized ordering of meaning thresholds, using the unconditional encompassing approach (Klugkist et al., 2005): a prior is placed over the full parameter space, but the evaluation focuses on the subset where the ordering constraint holds. The second model imposes no such constraint and allows the threshold parameters to vary freely.

To test the two hypotheses, we adopt model comparison methods using Bayes factors (Jeffreys, 1935; Kass & Raftery, 1995), the standard way to test hypotheses within the Bayesian framework. Appendix B provides details on the methodologies used to compute Bayes factors in the current study.

##### Robustness Checks and Computational Stability

Priors represent our initial beliefs or assumptions about the parameters in the model. However, even if the data are informative enough to overwhelm our initial beliefs, prior choices can greatly influence the model comparison results, which is one of the main challenges for Bayesian model comparison (e.g., Kass & Raftery, 1995). To determine the robustness of the Bayes factor, it is recommended to examine a range of plausible prior distributions and evaluate their effects on the results and conclusions (e.g., Sinharay & Stern, 2002; Vanpaemel, 2010; Lee & Vanpaemel, 2018). For our robustness tests, we focused on the group-level distributions of the main model parameters, that is, the group-level thresholds, vagueness, and response noise. For these parameters, we varied the width of the group-level mean (i.e., increase or decrease the range of plausible parameter values) and the width of the group-level variance (i.e., increase or decrease the homogeneity of individuals). The width of the group-level mean and variance was varied in two levels: narrow ($1/2 \times \sigma$) and wide ($2 \times \sigma$). For study 1, we additionally conducted a robustness check that focused on the correlation coefficients $\rho _\beta$ and $\rho _\gamma$. In the main model, the prior distribution mildly favored small to moderate correlations. To assess robustness, we additionally specified a prior that mildly favored higher correlations and therefore more strongly predicted data patterns consistent with the stability model. This high $\rho$ preferring prior for $\rho _\beta$ and $\rho _\gamma$ followed a $\text {Beta}(4, 2)$ which was stretched and shifted to a $[-1, 1]$ interval. In addition to the robustness checks, we also performed multiple computations of the Bayes factor to ensure estimation precision. For the main research questions and the specified statistical model, we calculated the Bayes factor twice. For the robustness tests, we computed the Bayes factors once. Parameter estimation and model comparison were based on a total of 100,000 samples from the prior and posterior distributions, with 1000 samples as burn-in.

### Results

**Fig. 4:**
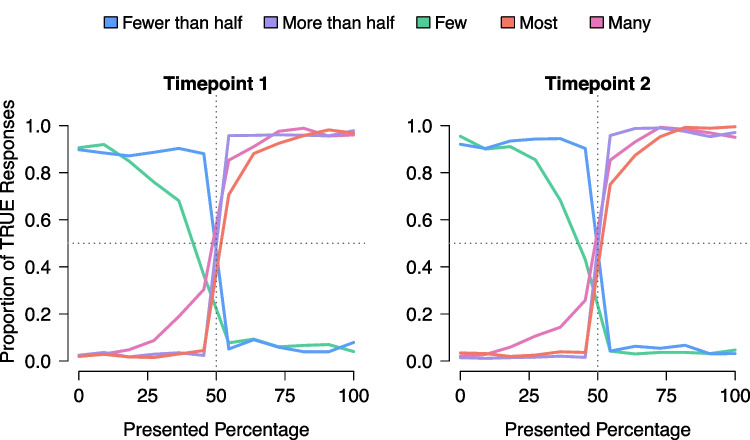
Observed response pattern for each quantifier and each time point, aggregated across all participants in study 1. The observed response pattern indicates differences in meaning between the quantifiers but only slight differences in meaning between the time points

**Fig. 5:**
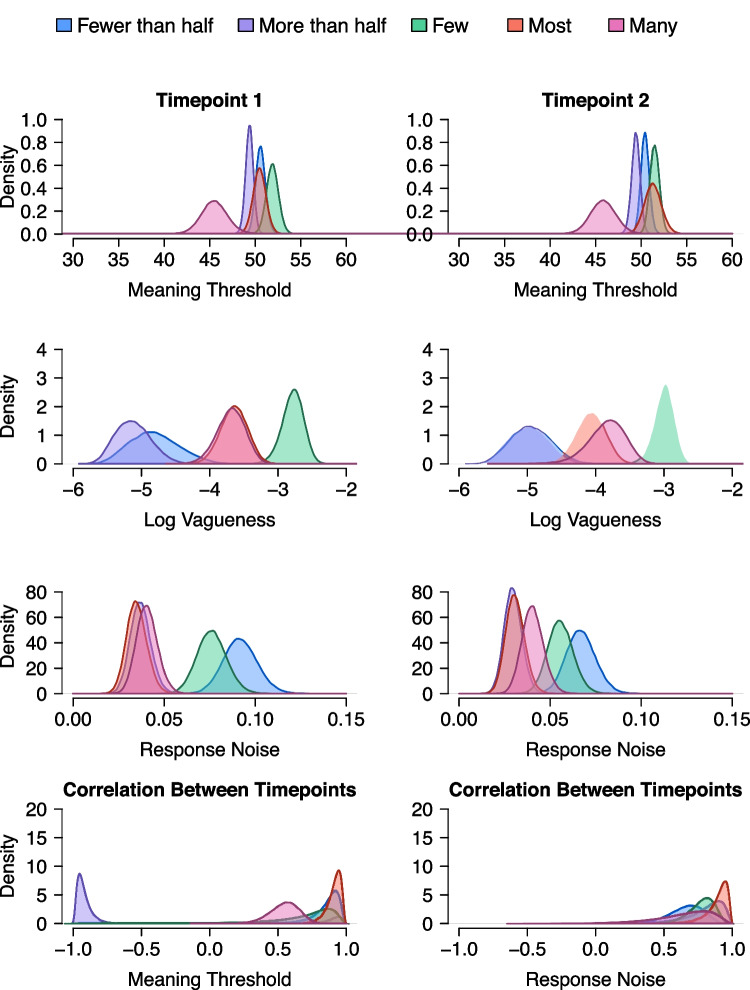
Group-level parameter estimates for each quantifier in study 1. The top three rows depict, for each time point, the meaning thresholds on its original scale, the vagueness parameter on the logarithmic scale (with values closer to zero indicating higher uncertainty around the meaning threshold), and the response noise estimates. The bottom row depicts the correlation estimates of the meaning thresholds and response noise between time points. For upward-entailing quantifiers, the meaning threshold marks the proportion above which the statement is considered TRUE; for downward-entailing quantifiers, it marks the proportion below which it is considered TRUE

Figure 4 presents the observed response patterns for each quantifier and time point, aggregated across all participants. The pattern highlights differences in semantic representation among the quantifiers while showing only minor differences between the time points. The group-level estimates for the meaning threshold (on its original scale), vagueness, and response noise are depicted in Fig. 5. The exact estimates are presented in Appendix C, Table 5. In the appendix, for the meaning threshold, we report both the estimate from the proposed model and the point at which the probability of considering a quantifier statement to be TRUE is 50%.

The relationship between time points is reflected in the moderate to high correlations between meaning thresholds across time, which aligns with the findings of Ramotowska et al. (2023). The highest correlation between thresholds is estimated for *most* with 0.92 and *many* with 0.55. However, the correlations for the quantifiers *more than half*, *fewer than half*, and *few* could not be reliably estimated, a challenge also noted in their paper for *more than half* and *fewer than half*. This issue likely arises from limited variability in thresholds between participants (see Rouder et al. (2023) for a detailed discussion of the challenges of measuring correlations under conditions of low individual variability in cognitive tasks). For these quantifiers, the posterior distributions appear bimodal and their credible intervals for the estimates nearly span the entire scale. The correlation between response noise parameters across times were moderate to high with the upward-entailing quantifiers *more than half* and *most* yielding higher correlations, compared to the remaining quantifiers.

The meaning thresholds for *more than half* are $49\%$ for both time points. For *fewer than half* they are $51\%$ for time point 1 and $50\%$ for time point 2. For the quantifier *most*, the meaning threshold is $50\%$ at time point 1 and $51\%$ at time point 2. For *many* the threshold falls below $50\%$, that is, $45\%$ at time point 1 and $46\%$ at time point 2. The meaning threshold for *few* falls above $50\%$ with $52\%$ for time point 1 and $51\%$ for time point 2. Note that for upward-entailing quantifiers (*many*, *more than half*, *most*), the meaning threshold (top row) indicates the proportion above which the quantifier is considered TRUE. For downward-entailing quantifiers (*few*, *fewer than half*), the meaning threshold indicates the proportion below which the quantifier is considered TRUE.

The results are largely consistent with the findings of Ramotowska et al. (2023), with the exception of the quantifiers *most*, which is estimated to have a substantially higher meaning threshold of approximately $65\%$ at both time points, and *few*, which is estimated to have a substantially lower threshold of around $23\%$ at both time points. These differences are likely due to the use of different cognitive models, specifically, the drift diffusion model in Ramotowska et al. (2023) versus the Bayesian hierarchical three-parameter logistic regression model used in the current study. Notably, analyses using a similar model in Ramotowska et al. (2024) yield parameter estimates comparable to those found in the present reanalysis.

The quantifiers *more than half* and *fewer than half* exhibit highly similar and low levels of vagueness, with this similarity increasing at time point 2. In contrast, *most* and *many* have similar levels of vagueness at time point 1 but become more dissimilar at time point 2, since the vagueness of *most* decreases while that for *many* remains stable. The quantifier *few* is associated with the highest uncertainty around the meaning threshold, which also remains stable.

In terms of response noise, the model estimates suggest a clear distinction between upward-entailing and downward-entailing quantifiers. Upward-entailing quantifiers show low response noise parameters at both time points, ranging between 0.04 and 0.03, while downward-entailing quantifiers show response noise parameters, ranging between 0.06 and 0.09. While response noise for *fewer than half*, *more than half*, and *few* slightly decreases at time point 2, it remains stable for *many* and *most*.

#### Hypothesis Testing

In this reanalysis, we studied the stability of semantic representation over time. The results of the reanalysis, that is, the log Bayes factors relating to both hypotheses are summarized in Table 1. We received overwhelming evidence in favor of a model that allows for changes over time; $\text {BF}_{12} = \text {exp}(\text {logBF}_{12}) > 1 \times 10^{304}$, which stands in stark contrast to Ramotowska et al. (2023) findings, who reported moderate evidence in favor of the null hypothesis that there are no differences in quantifier thresholds between sessions. We further conducted an exploratory analysis to assess this hypothesis for each quantifier separately. We found overwhelming evidence in favor of instability for all quantifiers; *more than half*, $\text {BF}_{12} = 9.65 \times 10^{34}$; *fewer than half*, $\text {BF}_{12} = 1.15 \times 10^{63}$; *few*, $\text {BF}_{12} = 7.47 \times 10^{63}$; *many*, $\text {BF}_{12} = 4.14 \times 10^{87}$; *most*, $\text {BF}_{12} = 2.17 \times 10^{61}$. As a caveat it should be noted that this exploratory result was fragile over different specifications of parameter priors for *more than half* and *fewer than half*; our hypothesis was confirmed for our main analyses and the model with narrow parameter priors and a high $\rho$ preferring prior. However, we receive evidence against our hypothesis for the model with wide parameter priors. For the quantifiers *few*, *many*, and *most* the results were stable over repeated computations and different specifications of parameter priors.

The relative ordering of the meaning thresholds within each individual, however, remained stable. We received overwhelming evidence that the ordering of meaning thresholds are stable over time for all individuals $\text {BF}_{12} = \text {exp}(\text {logBF}_{12}) = 4 \times 10^{24}$. The results of the main analyses are consistent over repeated computations. For hypothesis $\mathcal {H}_{1,1}$ we yield qualitatively identical results over our robustness conditions with different prior distributions. Hypothesis $\mathcal {H}_{1,2}$ was fragile over different specifications of parameter priors; for our main analysis and the model with wide parameter priors, our hypothesis is confirmed, whereas we receive evidence against our hypothesis for the model with narrow parameter priors.

**Table 1:** Log Bayes factors in favor of the hypothesis of interest ($\mathcal {H}_1$) compared to the alternative hypothesis ($\mathcal {H}_2$) for study 1 based on two Bayes factor computations, along with results from robustness checks to assess whether the conclusions hold under narrower or wider specifications of the prior distributions for the group-level parameters. See the main text for explanations regarding the competing hypotheses

	Main Analysis		Robustness Checks		
Hypothesis of Interest	Rep. 1	Rep. 2	Narrow Prior	Wide Prior	High $\rho$ Prior
$\mathcal {H}_{1,1}$	715.80	748.92	1,149.48	16.50	767.39
$\mathcal {H}_{1,2}$	56.64	57.02	-18.80	103.55	75.91

*Note.* A log Bayes factor higher than 2.3 indicates strong evidence ($\text {BF}_{12}>$ 10) in favor of the hypothesis of interest. A log Bayes factor higher than 4.6 indicates overwhelming evidence ($\text {BF}_{12}>$ 100) in favor of the hypothesis of interest. Negative log Bayes factors indicate evidence in favor of the alternative hypothesis

#### Posterior Predictive Distribution

**Fig. 6:**
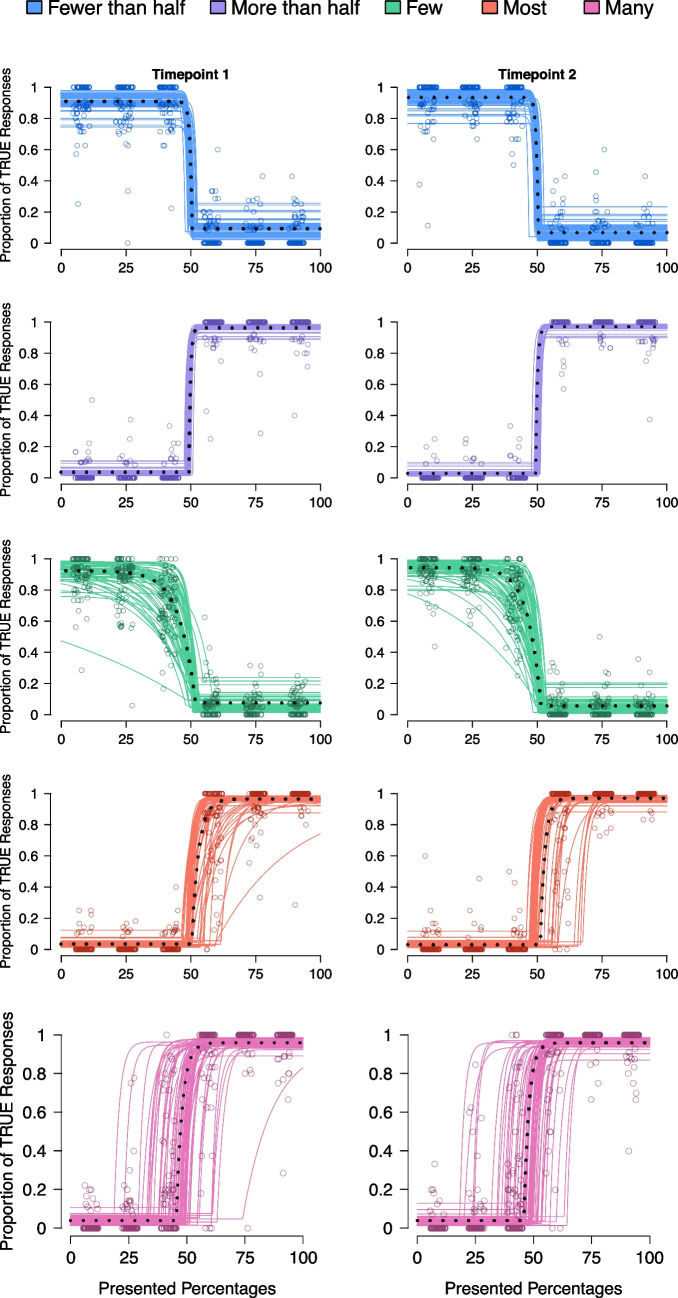
The figure shows the predicted response probabilities (solid lines) and observed response proportions (circles) for each quantifier and time point at the individual level in study 1. The group-level prediction is represented by the dashed line. The overlap between the predicted and observed response patterns suggests that the computational model is successful in re-describing the observed response behavior

The posterior predictive distribution can be interpreted as the model’s attempt to re-describe the behavioral data. A descriptive, adequate model should be able to capture the individual differences in individual behavior, including both extreme response patterns by some individuals and average ones by other individuals. Figure 6 shows the posterior predictive distribution on the group-level data for each quantifier and each time point. The figure suggests that the model is able to capture the response pattern quite well in both time points.

### Summary

Study 1 aimed to reanalyze data from experiment 2 in Ramotowska et al. (2023). The general patterns are comparable to those reported in their study, although some discrepancies likely stem from the use of a different cognitive modeling framework. In addition, we tested the stability of parameters over time using an analytical strategy that, in our view, is better suited to test the hypothesis of interest than the approach adopted by Ramotowska et al. (2023). While the original authors conducted separate Bayesian *t*-tests for each cognitive parameter and based their conclusions on individual results, our model evaluates all parameters simultaneously by comparing a model that allows correlations across time points with one that assumes all parameters are equal–thus reflecting the assumption that stability should apply to the cognitive model as a whole, rather than to isolated components. Consequently, even high correlations across time points in either the meaning threshold or response noise parameters do not, by themselves, imply stability: even small shifts in parameter values, across any component of the semantic model, can render the model that allows for change over time better able to account for the observed data than the model assuming identical parameters across sessions. In contrast to the conclusions of Ramotowska et al. (2023), who found moderate evidence that semantic representations remain stable over time, our reanalysis provides overwhelming evidence against the stability hypothesis.

While parameter estimates suggests that these changes are subtle, our hypothesis test strongly favors a statistical model that explicitly models change over time, rather than one that assumes the cognitive parameters from both experimental sessions are exactly equal. In addition, we found that although parameters vary across time points, the data suggest that the *ordering* of meaning thresholds remains stable within each individual: if an individual associates, for instance, *many* with smaller percentages than *more than half* and *most*, this ordering persists across both experimental sessions.

## Study 2: Stability Across Paradigms

In study 2, we conducted an experiment to explore the differences between model parameters across two paradigms: the linguistic and the visual version of the sentence-verification paradigm. Our aim was to reveal differences between visual and numerical processing arising from perceptual difficulties and to examine their influence on the vagueness and meaning-threshold parameters. Based on the research question, “Does the visual paradigm differ from the linguistic paradigm in terms of vagueness and between-subject variability in the thresholds?”, we derived the following hypotheses:

- $\mathcal {H}_{2,1}$: For every quantifier, we predict higher between-subjects variability in thresholds of the quantifiers in the visual paradigm than in the linguistic paradigm. The alternative hypothesis predicts that the variability between subjects in the threshold is equal in the visual and linguistic paradigm.
- $\mathcal {H}_{2,2}$: For every quantifier, there is more vagueness in the visual paradigm than in the linguistic paradigm. The alternative hypothesis predicts that vagueness is equal in the visual and linguistic paradigm.

### Method

#### Participants

Two hundred forty-eight individuals were recruited via Prolific, and 225 individuals completed both sessions. After individuals completed both sessions, we paired these data (i.e., an individual’s data was matched based on their (Prolific) worker IDs) and subsequently excluded responses with peculiar patterns based on the blinded data. Based on this evaluation, 9 out of the 225 individuals were excluded based on peculiar patterns. Twenty-seven individuals were excluded due to difficulties in pairing data from the two sessions, which corresponds to 16 percent of the sample. The high number of exclusions when matching task data occurred since individuals were required to manually enter their worker IDs before starting each session, which resulted in 27 individuals lacking corresponding entries. From the remaining sample, for 189 individuals, we removed 21 quantifier conditions from the data. Finally, 11 individuals failed the attention check, leaving us with a final sample of 178 participants.

After pairing task data and removing peculiar cases from the dataset, we determined the cutoff criteria for fast and slow responses. Based on the visual inspection of the response time data, we excluded trials in which individuals reacted faster than 500ms for each quantifier for both paradigms. We decided against excluding slow responses from the data due to the substantial variability in response times both among individuals and across quantifiers, which made it impractical to establish a meaningful exclusion criterion that did not affect the quality of the data. From all 57936 trials in the linguistic paradigm, we excluded 52 fast responses, which corresponds to 0.09 percent of all trials. From all 58428 trials in the visual paradigm, we excluded 56 fast responses, which corresponds to 0.1 percent of all trials.

##### Eligibility Criteria

Individuals had to meet the following criteria to be eligible for participation, namely, that (1) they had to be at least 18 years old to participate, (2) their first language was English, (3) they did not participate in our pilot study, (4) they indicated no color blindness, and (5) they indicated that they do not suffer from epilepsy or seizures. In addition, individuals were required to conduct the experiment on a computer or laptop; participation via mobile phones and tablets was not possible.

#### Design

**Fig. 7:**
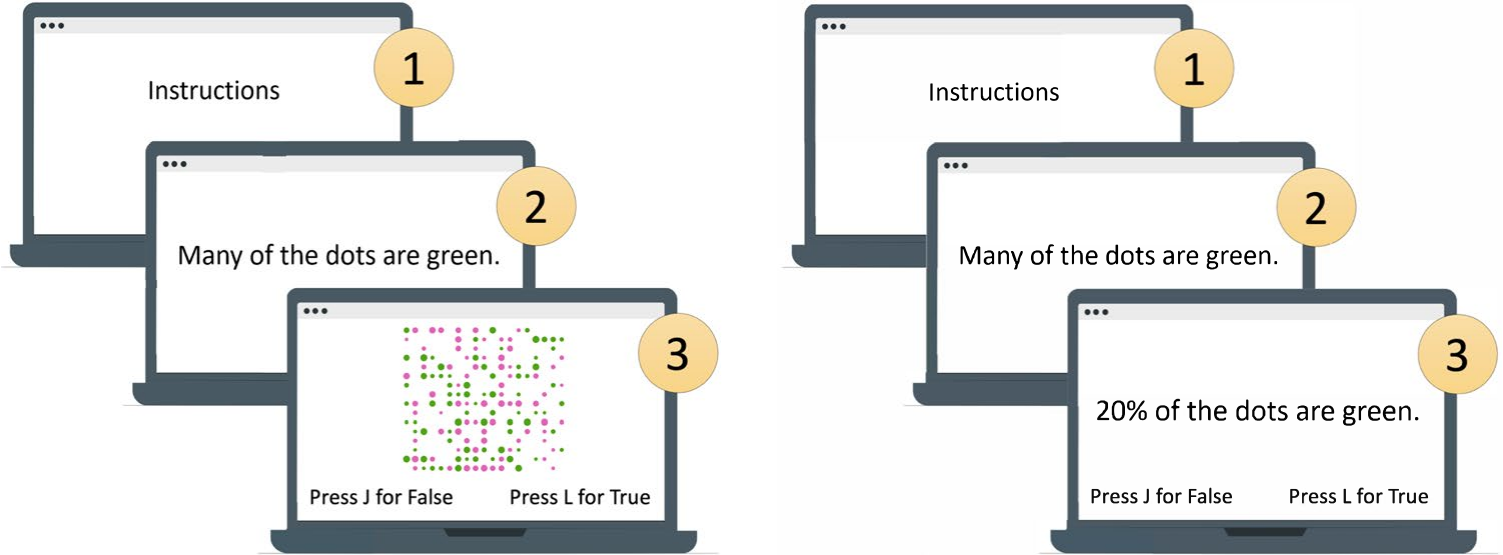
Schematic illustration of the experimental paradigm in study 2. In the visual paradigm (left), participants first read a quantifier statement, then viewed a dot pattern with two different colors, and finally judged whether the sentence and the picture matched. In the modified linguistic paradigm (right), participants first read a quantifier statement, then a statement containing a percentage, and judged whether the statements matched. In contrast to the linguistic paradigm in study 1, the statements in the modified linguistic paradigm referred to dots and colors rather than pseudowords. Participants completed both tasks approximately one week apart

These linguistic and visual versions of the sentence-verification paradigm can be considered conceptual replications of the sentence-verification paradigm used in study 1 by Ramotowska et al. (2023). Specifically, we implemented a visual version of the sentence-verification paradigm and a slightly adapted linguistic version of the sentence-verification paradigm. In the visual paradigm, individuals were presented with pairs of quantifier statements (e.g., “*Most*/ *Many*/ *Few*/ *More than half*/ *Fewer than half* of the dots are green.”) and visual stimuli (dot patterns of two different colors). Individuals then judged whether the quantifier sentence was TRUE or FALSE based on the presented dot pattern. In the modified linguistic paradigm, we replaced the pseudo-nouns and pseudo-adjectives with words describing dots and colors to align the linguistic paradigm with the visual paradigm. An example of a quantifier statement would be “*Most*/ *Many*/ *Few*/ *More than half*/ *Fewer than half* of the dots are green.”), an example of a statement containing a percentage would be “20% of the dots are green.”. The schematic illustration for a single trial for both tasks in study 2 is presented in Fig. 7.

The task order in study 2 was counterbalanced, that is, individuals performed either the visual or the linguistic paradigm first. Additionally, the key binding of the TRUE and FALSE judgments across individuals was counterbalanced. While the experimental stimuli were kept the same for all individuals, the presentation order of the experimental blocks and the presentation order of the stimuli within each experimental block was randomized across individuals.

#### Stimuli

The visual stimuli consisted of 432 dots randomly arranged on a 35 times 25 grid with two colors, inspired by the visual stimuli used in van Tiel et al. (2021). The presented percentages in both tasks were randomly drawn from normal distributions, mildly favoring values around the expected thresholds. More stimuli were presented around the expected thresholds to increase the number of informative trials around the threshold and, thus, to estimate the model parameters as accurately as possible.

The following means and standard deviations were used to draw the percentages in both tasks: *Few* (*M*=40, *SD*=20) / *Fewer than half* (*M*=50, *SD*=20)/ *More than half* (*M*=50, *SD*=20)/ *Many* (*M*=50, *SD*=30)/ *Most* (*M*=60, *SD*=30). For the visual paradigm, we colored the dots on the dot pattern based on the percentages drawn. For the modified linguistic paradigm, the percentages were rounded to the next integer. Percentages ranged from 1% to 99%, excluding 50%.

#### Procedure

Individuals were familiarized with the visual and linguistic sentence-verification paradigm by first completing a practice block of 12 trials. Individuals then completed three experimental blocks consisting of 104 trials (i.e., 312 trials in total), with each block corresponding to a different condition. In the visual paradigm, experimental conditions referred to the presented dot patterns which were colored in combinations of brown/purple, green/pink, or orange/blue. Moreover, the direction of the quantifier statements in the visual paradigm varied across trials (e.g., “Most of the dots are green.” vs. “Most of the dots are pink”), such that either color in each combination could serve as the target. In the linguistic paradigm, experimental conditions indicated sentences which referred to the colors brown, green, or orange. Individuals completed 60 trials per quantifier, with 20 trials per quantifier in each of the three color conditions, and 12 catch trials (using the quantifiers *all* and *none*) to assess attention. The visual and linguistic paradigms were conducted approximately one week apart. The median completion time for each paradigm was under 30 minutes, that is, approximately 22 minutes for the linguistic paradigm and 27 minutes for the visual paradigm.

#### Data Analysis

To test the differences in cognitive parameters across tasks, we fitted the data to our statistical model, which directly incorporates the potential relationship between tasks using the same covariance structure as in study 1. To test the first hypothesis, we computed a Bayes factor for the hypothesis that there is greater between-subjects variability in the meaning thresholds in the visual paradigm, compared to the hypothesis that the ordering of between-subjects variability in the tasks cannot be determined. Specifically, we first computed a Bayes factor which quantifies the predictive adequacy of a model in which the hypothesized ordering is adhered to–evaluated using the encompassing prior approach (Klugkist et al., 2005)–with a model that imposes no such constraint and allows parameters to vary freely. Next, we computed the Bayes factor for the hypothesis that the between-subjects variability in meaning thresholds is exactly equal in both paradigms, compared to the hypothesis that the ordering of between-subjects variability in the tasks cannot be determined. Finally, we tested the hypotheses of interest by multiplying the two Bayes factors. The second hypothesis was tested in the same way for the vagueness parameter. As in study 1, we tested whether our results are computationally stable and robust to different specifications of the prior distribution.

### Results

**Fig. 8:**
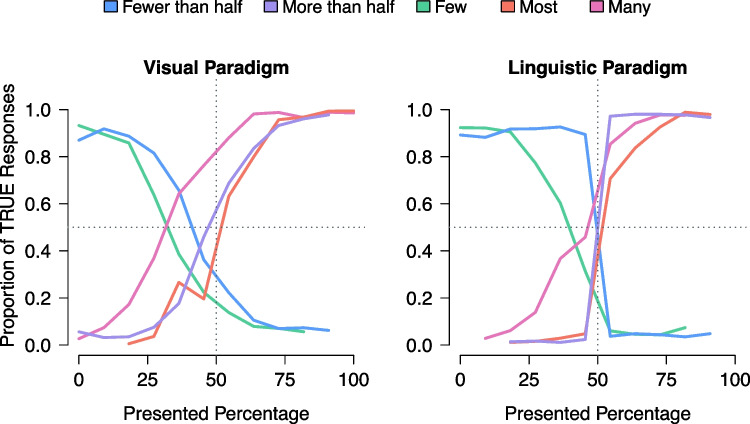
Observed response pattern for each quantifier and each paradigm, aggregated across all participants in study 2. The observed response pattern indicates differences in meaning between the quantifiers and differences in semantic representation between paradigms. Specifically, the observed response patterns in the visual paradigm suggest more vagueness across all quantifiers

**Fig. 9:**
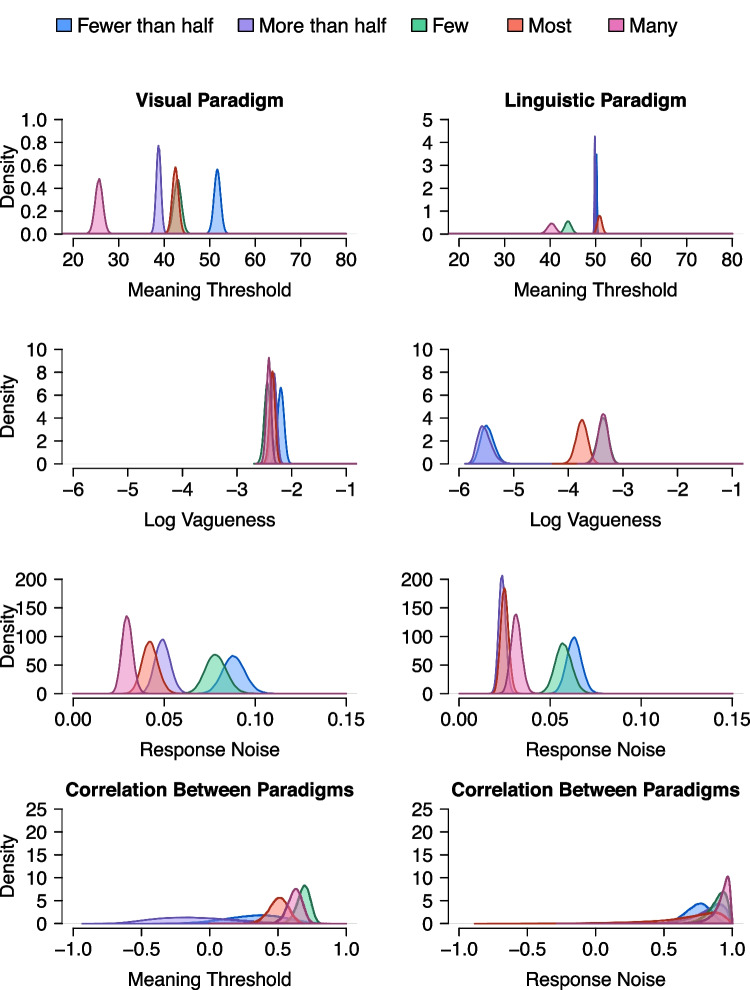
Group-level parameter estimates for each quantifier in study 2. The top three rows depict, for each paradigm, the meaning thresholds on its original scale, the vagueness parameter on the logarithmic scale (with values closer to zero indicating higher uncertainty around the meaning threshold), and the response noise estimates. The bottom row depicts the correlation estimates of the meaning thresholds and response noise between paradigms. For upward-entailing quantifiers, the meaning threshold marks the proportion above which the statement is considered TRUE; for downward-entailing quantifiers, it marks the proportion below which it is considered TRUE

Figure 8 presents the observed response patterns for each quantifier and paradigm, aggregated across all participants. The pattern highlights differences in meaning among the quantifiers and major differences between the paradigms. The group-level estimates for the meaning threshold (on its original scale), vagueness, and response noise are depicted in Fig. 9. The exact estimates and detailed summaries for the group-level estimates for the meaning threshold are presented in Appendix C and Table 6. In the appendix, for the meaning threshold, we again report both the estimate from the proposed model and the point at which the probability of considering a quantifier statement to be TRUE is 50%.

The correlation between thresholds in both paradigms is moderate to high for the quantifiers *few*, *many*, and *most*, with correlations above 0.51. As in study 1, the correlation between thresholds for the quantifiers *fewer than half* and *more than half* was low and included negative values, which we attribute again to the lack of individual variability in the linguistic paradigm. The posterior distributions for these parameters do not appear bimodal (as in study 1); however, they are flat, suggesting that the data may not have sufficiently updated the prior.

The correlations between response noise rates were high for all quantifiers, with all correlations exceeding 0.73. The highest correlation was found between response noise across paradigms for the quantifier *many*, followed by *few*, *more than half*, *fewer than half*, and finally *most*, which featured a 95% credible interval that included negative correlations. When interpreting the correlation parameters, it is important to note that, since paradigm and time were confounded in study 2, the observed correlations may reflect differences between the visual and linguistic paradigms, but also time-related changes, or a combination of both. Thus, the correlation parameters represent the extent to which individual differences in meaning thresholds and response noise are preserved across sessions, but in the current design they do not allow us to isolate the underlying source of (in)stability.

We also found substantial differences between meaning thresholds in the visual and linguistic paradigm. That is, individuals at the group level adopted lower thresholds in the visual paradigm quite dramatically for upward-entailing quantifiers *more than half* ($50\%$ versus $39\%$), *most* ($51\%$ versus $42\%$), and *many* ($40\%$ versus $26\%$). For downward-entailing quantifiers the shift was less dramatic and went in opposite directions. While individuals adopted lower thresholds in the visual paradigm for the downward-entailing quantifier *fewer than half* ($50\%$ versus $48\%$), individuals, at the group level, adopted higher thresholds for *few* ($57\%$ versus $56\%$). Individuals had difficulties accurately estimating the percentages of the relevant stimuli in the visual paradigm. Interestingly, these difficulties resulted in inconsistent estimation patterns, such as the seemingly non-monotonic response pattern for *most*, rather than a systematic tendency to over- or underestimate of percentages.

Differences in meaning thresholds between the two paradigms are also reflected in considerable differences in vagueness. As expected, we found higher vagueness estimates for all quantifiers in the visual paradigm compared to the linguistic paradigm. Interestingly, the vagueness estimates in the visual paradigm are nearly identical across all quantifiers and center around $-2.3$. For the linguistic paradigm, the vagueness estimates cluster together for quantifiers with vague and sharp meaning thresholds. That is, the median of the vagueness parameters for the quantifiers *fewer than half* and *more than half* are estimated to be about $-5.49$ and 5.55, respectively. For *most*, the median estimate is $-3.76$. The quantifiers *few* and *many* have identical estimates of $-3.37$. Note that the ordering of the vagueness parameters in the linguistic paradigm does not align with the results from study 1, where we found higher vagueness for *few* compared to *many*.

The ordering of response noise parameters in the linguistic paradigm, on the other hand, does align with that found in study 1. Specifically, we observed the lowest response noise for *more than half*, followed by *most* and *many*. We also replicated the counter-intuitive finding that *few* had a lower response noise rate than *fewer than half*. In the visual paradigm, we similarly found that upward-entailing quantifiers had lower response noise rates than downward-entailing quantifiers. However, the two paradigms differ in the ordering among upward-entailing quantifiers: *many* had the lowest response noise rate, followed by *most* and *more than half*. Overall, response noise was higher across all quantifiers in the visual than in the linguistic paradigm, which may reflect task-specific demands, for instance, misreading of color terms due to variation in the direction of the quantifier statements.

**Fig. 10:**
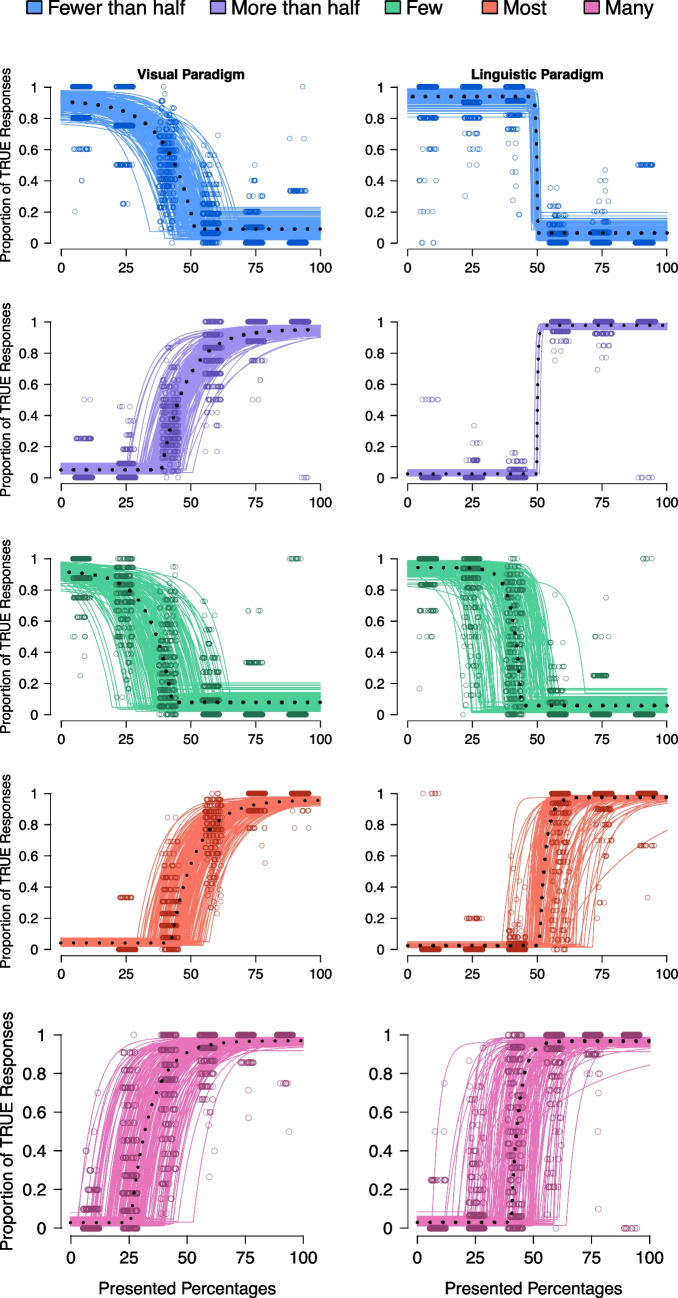
The figure shows the predicted response probabilities (solid lines) and observed response proportions (circles) for each quantifier and paradigm at the individual level in study 2. The group-level prediction is represented by the dashed line. The overlap between the predicted and observed response patterns suggests that the computational model is successful in re-describing the observed response behavior

Figure 10 displays the posterior predictive distribution of the group-level data for each quantifier and each paradigm. The figure suggests that the model is able to capture the behavioral data well for both the visual and the linguistic paradigm.

#### Hypothesis Testing

Results of the hypothesis tests are summarized in Table 2. Our research question addressed whether the visual paradigm would differ from the linguistic paradigm regarding the between-subject variability of the meaning threshold parameter and the vagueness of the meaning thresholds. We found overwhelming evidence supporting our predictions. Specifically, we hypothesized that each quantifier in the visual paradigm would be associated with higher between-subject variability in thresholds compared to the linguistic paradigm. We found overwhelming evidence in favor for this hypothesis, with $\text {BF}_{12} = \text {exp}(\text {logBF}_{12}) = 7.48 \times 10^{30}$. In addition, we hypothesized that each quantifier in the visual paradigm would exhibit higher vagueness than in the linguistic paradigm. This hypothesis too received extreme support, with $\text {BF}_{12} = \text {exp}(\text {logBF}_{12}) = 1.17 \times 10^{110}$. Results were robust against multiple repetitions and alternative plausible prior specifications.

**Table 2:** Log Bayes factors in favor of the hypothesis of interest ($\mathcal {H}_1$) compared to the alternative hypothesis ($\mathcal {H}_2$) for study 2 based on two Bayes factor computations, along with results from robustness checks to assess whether the conclusions hold under narrower or wider specifications of the prior distributions for the group-level parameters. See the main text for explanations regarding the competing hypotheses

	Main Analysis		Robustness Checks	
Hypothesis of Interest	Rep. 1	Rep. 2	Narrow Prior	Wide Prior
$\mathcal {H}_{2,1}$	71.09	73.69	71.01	71.48
$\mathcal {H}_{2,2}$	253.44	224.11	253.37	253.28

*Note.* A log Bayes factor higher than 2.3 indicates strong evidence ($\text {BF}_{12}>$ 10) in favor of the hypothesis of interest. A log Bayes factor higher than 4.6 indicates overwhelming evidence ($\text {BF}_{12}>$ 100) in favor of the hypothesis of interest

#### Exploratory Analysis: Trade-Off Between Vagueness and Response Noise

**Fig. 12:**
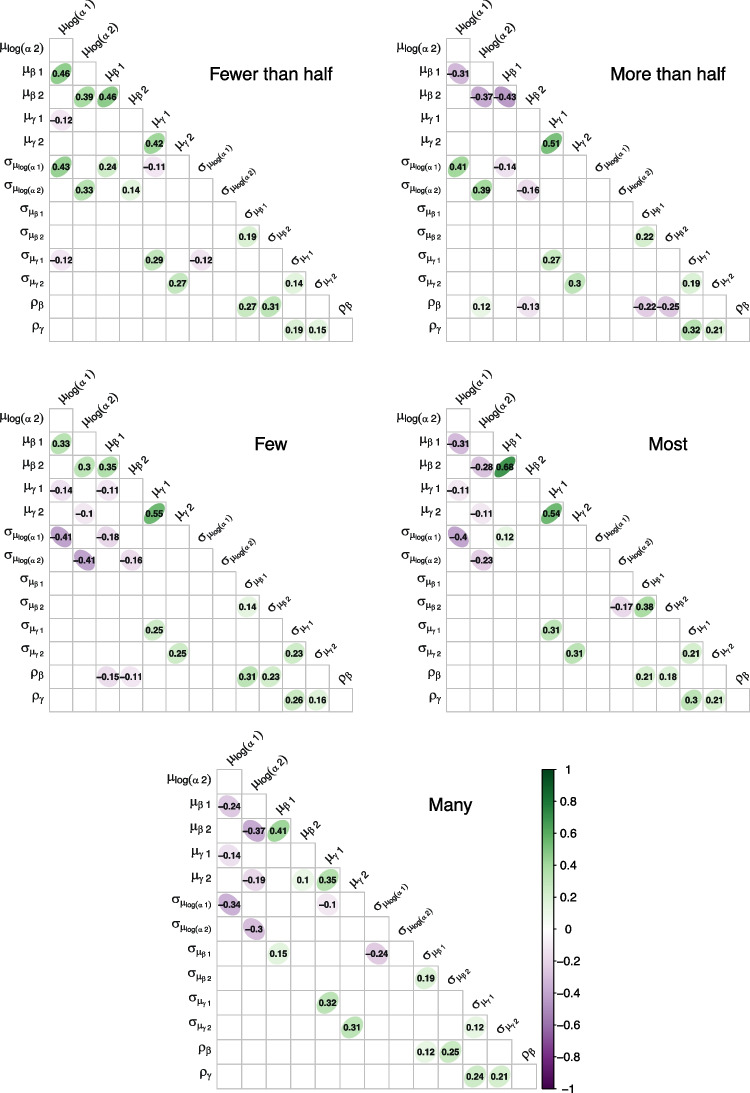
Spearman correlation coefficients between the group-level parameter estimates in study 1. Correlations above 0.1 are shown in green, those below $-0.1$ are shown in purple. The subscripts indicate whether the estimate refers to time point 1 or time point 2

**Fig. 14:**
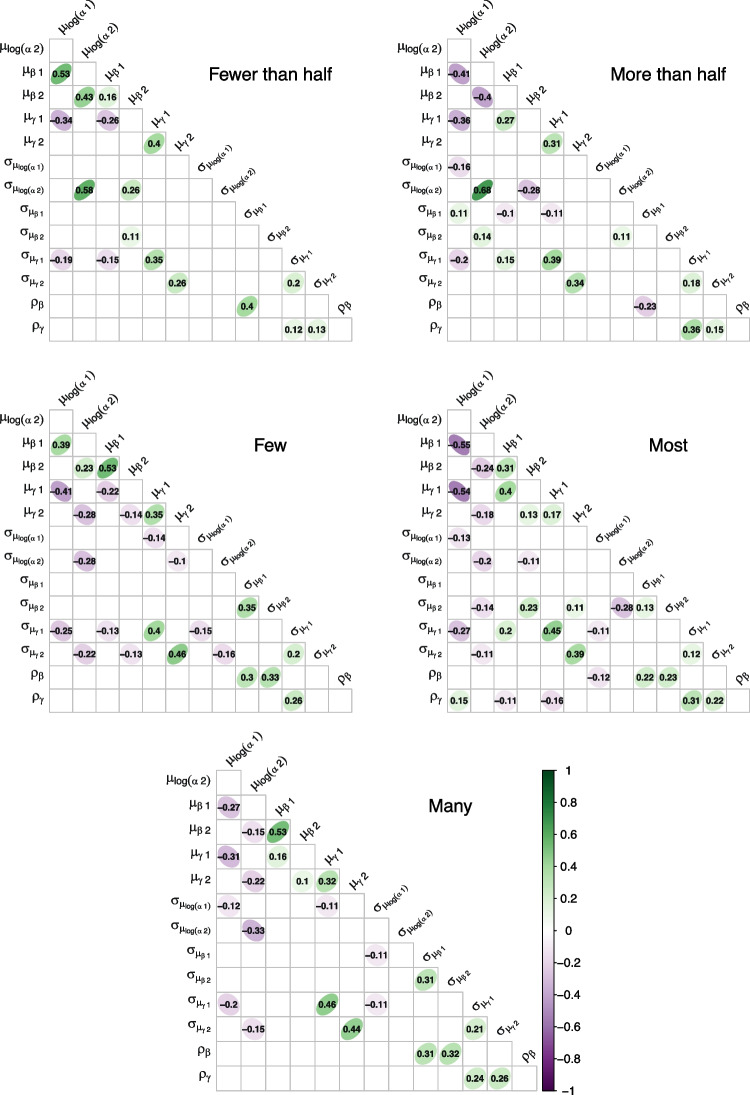
Spearman correlation coefficients between the group-level parameter estimates in study 2. Correlations above 0.1 are shown in green, those below $-0.1$ are shown in purple. The subscripts indicate whether the estimate relates to the visual (1) or linguistic (2) paradigm. In the visual paradigm, for each quantifier, response noise $\mu _{\gamma ,1}$ is negatively correlated with vagueness $\mu _{\text {log}(\alpha ,1)}$ (in the range of $-0.31$ to $-0.54$)

The unusual pattern of vagueness parameters in the visual paradigm (i.e., the clustering of posterior distributions at the same value) raised concerns about possible model misspecification and thus prompted us to examine the correlation between the group-level estimates in both studies (Figs. 12 and 14). For study 1 and the linguistic paradigm in study 2, we did not observe systematic relationships between vagueness and other group-level parameters. The visual paradigm in study 2, on the other hand, showed negative correlations between response noise and vagueness for each quantifier. We therefore conducted further exploratory analyses to rule out the possibility of an undesired trade-off between the estimation of response noise and vagueness. A potential trade-off in the model could link the vagueness and response noise parameters which would make it harder to recover true vagueness, as its estimates may be influenced by response noise. The results of this exploratory analysis can be found in our online supplements. When fitting the modified version of our model which fixed response noise across paradigms, vagueness parameters in the visual paradigm still clustered around $-2.3$. Simulations from the prior distribution, with vagueness centered at $-2.3$, revealed no correlation between response noise and vagueness parameters. Moreover, our model validation confirmed that true parameter values could be successfully recovered when vagueness was high (i.e., centered at $-3$). Based on these results, we conclude that this relationship is not due to an inherent trade-off in our model but rather a consequence of the specific task demands.

### Summary

Study 2 aimed to compare quantifier meanings across paradigms using the visual and linguistic sentence-verification paradigm. Specifically, the study revealed predicted differences in semantic representations of quantifiers between the visual and linguistic paradigm. In the visual paradigm, we observed greater vagueness around the meaning threshold and more variability in meaning thresholds across individuals. Moreover, the correlation between vagueness and response noise in the visual paradigm suggests the presence of a task-specific source of error that warrants further investigation in future research.

## Discussion

When studying the meaning of words and expressions in experimental studies, researchers frequently use probabilistic information to complement existing logical theories and better capture the complexity of real-world data. One way to do this is by semantic representations, that is, statistical models that describe an individual’s response behavior with semantically meaningful parameters. In this study, we introduced a Bayesian hierarchical model that describes the semantic representation of quantifiers in terms of meaning thresholds, vagueness, and response noise in sentence-verification paradigms. This statistical model can be applied to study semantic representations across two time points and paradigms. Compared to its predecessor proposed by Ramotowska et al. (2024), this model effectively integrates the field’s rich prior knowledge and logic-derived assumptions, accounts for individual differences, and defines the meaning threshold as the necessary and sufficient condition for the truth of quantifier statements.

We demonstrated the model’s capabilities by applying it to two datasets to test the stability of semantic representation over time and across different paradigms. In study 1, we found evidence of changes in semantic representation over time, while the relative ordering of individual meaning thresholds remained stable. These findings suggest a revision of the assumption that meaning, when operationalized as semantic representation, is a stable, inherent property over time. Instead, our key dimensions of semantic representation, that is, meaning threshold, vagueness, and response noise, appear to be dynamic. Alternatively, this could suggest that the meaning threshold is not represented as an unambiguously value, but rather as a probabilistic distribution over a range of values.

In study 2, we revealed substantial differences between visual and linguistic paradigms. While the relative structure of meaning thresholds remained consistent within individuals (as indicated by study 1), the finding that vagueness around the meaning thresholds is greater in the visual paradigm compared to the linguistic paradigm further supports the approach that meaning representations are probabilistic constructs. Additionally, the correlation between vagueness and response noise in the visual paradigm suggests the presence of an additional source of error that could be contributing to differences in semantic representations. Since the linguistic and visual paradigms were administered one week apart, this raises the possibility of time-related effects similar to those observed in study 1. However, the lower correlations observed in study 1 and the absence of such correlations in the linguistic paradigm of study 2, together with our model validation analyses, suggest that the present relationship is more consistent with task-related influences than with time-related effects alone. Study 2 therefore highlights the importance of accounting for paradigm-specific influences on meaning representation and further complicates the assumption of stability. Future research should further explore the sources of variability across paradigms, particularly focusing on the task-specific influences on vagueness.

### Sources of Vagueness in Visual Paradigms

Several theoretically predicted patterns emerged across paradigms in study 2. In the linguistic paradigm, response noise was lower for upward-entailing than for downward-entailing quantifiers, and both vagueness and between-subject variability were higher for quantifiers with vague meaning thresholds than for those with sharp meaning thresholds. Moreover, in the visual paradigm, we observed higher response noise for downward-entailing quantifiers and greater between-subject variability for vague quantifiers. These selective patterns are consistent with the intended interpretation of the model parameters.

At the same time, the nearly identical vagueness estimates across quantifiers in the visual paradigm raise questions about the interpretation of the vagueness parameter, and whether the two paradigms are measuring the same semantic constructs. In the linguistic paradigm, vagueness signifies genuine uncertainty around the meaning threshold, whereas in the visual paradigm, difficulties in perceptual processing may have contaminated the vagueness parameter. That is, difficulties in estimating quantities may contribute not only to the shift in meaning thresholds but also to uncertainty around them. Regarding the quantifier *more than half*, for instance, we found that individuals seem to consistently underestimate the quantities and thus have lower meaning thresholds than one would expect.

Our findings on increased vagueness in the visual paradigm resonate with broader debates in numerical cognition on how magnitude and quantity are represented. In particular, Leibovich et al. (2016) and its accompanying commentaries (Sasanguie & Reynvoet, 2017; Gebuis et al., 2017) challenge the approximate number system (Dehaene, 1997) and support the idea of differences in perceptual processing in a visual versus a linguistic paradigm. This perspective is directly relevant to our finding that the participants showed a more variable (and imprecise) behavior in the visual paradigm compared to the linguistic one. Although we did not model number cognition parameters explicitly, future work could assess in detail how parameters, such as the “Weber Fraction” Inglis and Gilmore (2014), relate to semantic dimensions like vagueness or threshold placement. Another important direction for future research would be to disentangle more explicitly perceptual noise when estimating proportions from less consistent decision criteria, for instance, by adopting a signal detection theory framework (Green & Swets, 1966; Tanner & Swets, 1954). Our current decision to exclude these components was motivated by both scope and measurement limitations: the visual task was not designed to isolate number perception independently of semantic representation, and extending the model to include number-specific priors would have introduced considerable complexity. In contrast, quantifier judgments in the linguistic paradigm appear more stable, potentially because symbolic language facilitates retrieval and comparison processes (Elizabeth & Spelke, 2003; Feigenson et al., 2004).

As a result, an important direction for future research would be to investigate the sources of vagueness in the visual paradigm, particularly by manipulating the difficulty in perceptual processing. If vagueness in the visual paradigm is purely due to estimation noise and imprecision, reducing the number of dots in the visual pattern would likely decrease vagueness. If, however, vagueness in the visual paradigm arises from a different internal representation of quantities compared to the linguistic paradigm, reducing the number of dots might shift the meaning threshold in the visual paradigm closer to the linguistic one but may not reduce vagueness as effectively. In addition, previous research by Deschamps et al. (2015) showed that the polarity effect disappeared when symbolic verification statements (e.g., inequality signs flanked by colored squares) were used instead of linguistic ones. Building on this, future studies could investigate whether symbolic verification statements can contribute to reducing imprecision, and whether they influence only the response noise parameter or also affect vagueness.

### Application to Other Research Areas

While the current study focused on applying the proposed model to quantifier words, its framework is well-suited for studying semantic meaning more broadly. In particular, we believe that the computational model can also apply to capture different linguistic constructs, for instance, gradable adjectives (Johnson et al., 2026) or be used to test the universality of linguistic phenomena. A suitable candidate in this context is the monotonicity effect, which, as suggested by Schlotterbeck et al. (2020), is so robust that it may be inherent to language processing. Another prominent probabilistic model in the literature is the Rational Speech Act framework (i.e., RSA; Frank & Goodman, 2012; Franke, 2014; Qing & Franke, 2014), which models meaning through Bayesian pragmatic reasoning between speakers and listeners. In contrast to the current research, the RSA models how speakers select utterances and how listeners interpret them by using Bayesian reasoning to assess how informative they are in a given context. The strength of the present model is its focus on the semantic representation of quantifiers before adding the complexity of pragmatic considerations, which can vary across communicative contexts. Since the goal of the current study was to examine the stability or variation of quantifier meanings, we therefore investigated these representations independently of pragmatic influences. At the same time, future research could integrate the proposed computational model into communicative paradigms to study how pragmatic updating affects specific semantic parameters.

A more challenging, but potentially fruitful, extension would be the application of the framework to common nouns (Verheyen et al., 2010, 2019). For instance, research by Verheyen et al. (2019) suggests that category membership decisions for common nouns change over time due to participants adopting different criteria for membership, and Verheyen et al. (2010) report moderate stability of individuals’ decision criteria across noun categories. What makes the application of the proposed model more challenging in this context is that data from categorization paradigms differ structurally from those in quantifier research or studies on gradable adjectives. Unlike in quantifier research, where proportions can be systematically varied for the same expression, categorization tasks often involve a single binary membership judgment for each item (e.g., whether a *penguin* is a *bird*). While it is in principle possible to design continuous categorization tasks, for instance, by asking participants to place an item such as *penguin* on a scale between *bird* and *not bird*, standard paradigms usually rely on discrete judgments for individual items. From this perspective, a threshold for common nouns would not represent a proportion, but rather a decision criterion placed along a latent similarity or typicality dimension that determines category membership. Incorporating such a latent dimension into the present framework would therefore require a corresponding extension of the model architecture.

The current findings also have implications for research areas that assume stable semantic representations across time or experimental paradigms. The modeling approach used in this study captured time-variant parameters and suggested that both the meaning threshold and response noise may be dynamic across time and experimental paradigms. While differences in response noise could be attributed to learning effects (e.g., lower values at the second time point) or task-related factors (e.g., higher response noise in the visual compared to the linguistic paradigm), the sources of time-variance in the meaning threshold are less well understood. It may be that the meaning threshold should not be treated as a value that can be expressed unambiguously (cf. Heim & Kratzer, 1998; Coppock & Champollion, 2019; Douven et al., 2017), but rather as a probabilistic construct which warrants its formalization through probabilistic models, such as Bayesian cognitive models that represent thresholds as distributions and explicitly capture uncertainty around them, or hierarchical models that allow for systematic individual differences across participants. Further research is needed to better understand these differences and identify the factors driving these changes. Notably, despite these absolute changes, the relative ordering of meaning thresholds remained stable which suggests some robustness in the underlying semantic structure.

The differences between the visual and linguistic paradigm in study 2 also highlights another important application area of our model: confirming the construct validity of newly developed paradigms. That is, paradigms designed to capture the same semantic constructs should positively correlate and generally result in similar behavioral patterns. Thus, as paradigms related to semantic representation continue to evolve (e.g., through different modalities of presentation or different stimulus sets), the current model could serve as a valuable tool in establishing their validity.

Finally, the current model is suitable for studying quantifier processing in neurotypical individuals as well as in individuals with selective language deficits, and for evaluating the effectiveness of behavioral interventions (e.g., Bochynska et al., 2020; Prescott et al., 2024; Reißner et al., 2024). For instance, the experimental design used by Reißner et al. (2024) consisted of a baseline phase, an intervention phase using a feedback paradigm targeting the interpretation of *many*, and a subsequent test phase. Within our framework, this design could be analyzed to determine which semantic parameters are affected by the intervention and to assess improvements, for example through shifts in meaning thresholds in the intended direction or reductions in vagueness. The framework may also be applied to experimental manipulations that affect group dynamics (e.g., Murthy et al., 2022) or lexical disagreements (e.g., Martı et al., 2023; Waldon et al., 2023; ; Wang & Bi, 2021). More generally, researchers could investigate whether and how interventions or experimental manipulations (e.g., parent-mediated interventions or building a common ground with other group members) influence semantic representations, whether the different semantic parameters are impacted equally, or whether certain interventions specifically target particular parameters.

When applying the present modeling framework to other research areas, several design considerations should be taken into account. First, precise parameter estimation benefits both from a sufficiently large number of trials per participant and from careful selection of target items. Increasing the number of trials per individual generally improves the precision of parameter estimates and can also stabilize group-level quantities (e.g., correlation coefficients). In the present study, this was reflected in the greater precision of the correlation estimates in study 2 (60 trials per participant and quantifier) compared to study 1 (50 trials per participant). However, researchers should also consider characteristics of their target items, such as potentially limited between-subject variability, which can hinder effective updating of prior distributions even when the number of trials is large. These considerations become particularly relevant in research contexts where administering a large number of trials is not feasible, for instance, in clinical or developmental populations.

### Adaptation of the Model Assumptions Based on Research Applications

The model is built on several key assumptions that can be relaxed or adjusted depending on its application. Currently, the model is designed for single bounded quantifiers, which involve a single meaning threshold (e.g., *more than half*). If researchers aim to model response probabilities for quantifiers with multiple thresholds (e.g., *about half*; *some, but not all*), or different interpretations within populations (e.g., some interpreting *more than half* as *more than 50%* and others interpreting it as *more than half, but not all*), fundamental changes to the model would be required. To accommodate more complex quantifiers or a combination of different populations, a future version of this model may require a different, more flexible link function and mixture components, which requires extensive research and validation. However, other key assumptions can be adjusted in a more straightforward fashion.

An assumption that can be easily relaxed is that individuals perform no worse than chance. While this is a reasonable assumption for research on healthy adults, different research areas, such as clinical research, may want to relax this assumption. For instance, Bochynska et al. (2020) studied differences in spatial language performance between typically developing adults and highly functioning adults on the autism spectrum and found group differences for projective prepositions (*left*/*right*, *front*/*back*), proximal terms (*near*/*far*), and source path terms (*out of*/*down off*/*away from*). If our model were to be used in such contexts to study selective deficits, the truncation on the response noise parameter could be adjusted to account for a wider range of behavioral patterns, including worse than chance performance.

Another modeling assumption concerns the constant (symmetric) response noise rate. In the present model, the response noise parameter affects responses both below and above the meaning threshold to the same extent, implying that lapses are equally likely to produce false alarms (i.e., TRUE responses below the meaning threshold) and misses (i.e., FALSE responses above the meaning threshold). Consequently, the model assumes that error rates are symmetric around the threshold. However, systematic response biases, such as a general tendency to respond TRUE, may generate asymmetric error patterns (e.g., more false alarms than misses) that cannot be represented by a single symmetric noise parameter and may lead to spurious shifts in the estimated meaning threshold parameter. Allowing for asymmetric error rates would make it possible to distinguish directional response biases from genuine shifts in meaning threshold parameters. However, a model incorporating both vagueness and asymmetric error rates may not be identifiable.

Furthermore, the present model accounts for relationships between time points or paradigms within each quantifier. To capture these relationships, we decomposed the covariance matrix in the model into correlations and standard deviations and placed separate prior distributions on each. While this decomposition is appropriate for comparisons involving two time points or paradigms, it becomes insufficient when extending the model to more than two, for instance, to three, five, or ten time points. In such cases, it is necessary to restructure the model and place a prior directly on the full covariance matrix, by decomposing it, for instance, into a vector of standard deviations and a Cholesky-factorized correlation matrix for which the LKJ distribution is a suitable prior (for computational details and an example application, see Development Team (2022) and Sarafoglou et al. (2024)).

Through this advancement, the proposed computational model could be used to study changes in semantic representation over longer periods of time or to systematically investigate it for single quantifier across multiple paradigms. For instance, Register et al. (2020) showed that individuals’ performance in truth judgments tasks for *most* depends on different verification strategies that are task-sensitive, varies between individuals, and is influenced by factors such as the number of trials. While (Register et al., 2020) focused on the proportion of TRUE responses and response time differences, our computational model could help identify relationships between paradigms across the three proposed parameters.

Relatedly, our model could feature a shared underlying structure not only among time points or paradigms, but also among model parameters across quantifiers as well as dependencies among different parameters within a given quantifier. Such an extension would relax the independence assumption between quantifiers, which has been questioned in previous literature (e.g., Ramotowska et al., 2024; Hackl, 2009; Carcassi et al., 2021; Heim et al., 2020) and would introduce a structured dependency among semantic components. To do so, the model architecture would need to be extended with an additional covariance matrix across the parameters of interest. The prior structure used for generalizing the model to multiple time points or paradigms could also be applied in this case. Such an extension would allow researchers not only to systematically investigate effects across time/paradigms, but also to test, for instance, whether individuals who adopt relatively higher meaning thresholds for one quantifier (e.g., *most*) also tend to adopt higher thresholds for other quantifiers, which might reflect a general tendency to require relatively higher or lower proportions before judging quantifier statements as true. Researchers could also study relationships between vagueness and response noise parameters, or between vagueness and meaning thresholds, to test more specific hypotheses about the structure of semantic representations.

The proposed model uses an exponential link function to relate response behaviors to presented percentages and interprets the meaning threshold as the necessary and sufficient condition for the truth of a quantifier statement. This meaning threshold, however, may not always align with intuitive interpretations. For instance, the meaning threshold for *few* was estimated to be 52% (study 1) or 57% (visual paradigm, study 2), even though a prototypical percentage may be associated with much smaller values. Thus, depending on the study goal, researchers may also consider the point at which the proportion of TRUE responses reaches 50%, which aligns with the interpretation of the threshold in traditional computational models using a logistic link function. Importantly, in the present paradigms, the exponential and logistic link functions yield highly similar predictions and can hardly be distinguished on the basis of their observable response patterns; their primary difference lies in the interpretation of the parameters rather than in model fit. Although we did not formally compare the two specifications, such comparisons may be informative in contexts where theory does not favor one over the other, particularly when experimental paradigms provide sufficiently fine-grained data around the meaning thresholds to distinguish between them.

## Conclusion

Our findings challenge the assumption that semantic representations of logical vocabulary have stable, fixed values. Instead, they suggest that these representations are probabilistic constructs. At the same time, our results reveal a different kind of stability: the relative ordering of these representations remains consistent within individuals. Taken together, these findings indicate that researchers in experimental semantics should reconsider the assumption of stability in the meaning of logical terms. Instead, our work points to a promising direction for future research, one that focuses on the relative stability of meaning representations within individuals, redefined in terms of the ordering of meaning thresholds. Bayesian hierarchical models offer an effective framework for capturing these nuances, as they explicitly model individual-level effects, allow domain-specific expertise to be formalized through prior distributions, and enable the use of Bayes factors to test for specific orderings. Moreover, the versatility of the model allows for the description of semantic representation in a broad spectrum of linguistic constructs. The present work provides a basis for future developments in computational modeling within the field of computational semantics.

## Disclosures

### Preregistration and Analysis Blinding

Prior to conducting the analysis for our case study, we preregistered the full procedure on the Open Science Framework at https://doi.org/10.17605/OSF.IO/5ZP7Q (for study 1) and https://doi.org/10.17605/OSF.IO/2RAZV (for study 2). These analyses were then verified and adjusted –if necessary– based on the blinded version of the data. The final analysis pipeline for both studies was uploaded to the OSF project page before the analysis on the real data was carried out. Note that the main body of the paper presents a subset of the preregistered hypotheses. We report the results for the hypotheses directly related to the benefits of the proposed statistical model, particularly its ability to test the stability of meaning representation. Other preregistered hypotheses focused on verifying data patterns reported by Ramotowska et al. (2023) and Ramotowska et al. (2024), confirming the existence of vagueness at the individual level, and examining differences between *most* and *more than half*. These remaining hypotheses and their corresponding results can be found in Appendix A.

#### Deviations from the Preregistration

For study 1, the implementation of the computational stability and the computational details (i.e., the number of samples) deviated from the preregistered analysis plan. That is, we preregistered to draw a total of 250, 000 samples to compute the Bayes factor. In addition, we planned to repeat the Bayes factor computation for the specified statistical model 5 times and for the robustness tests three times, thereby presenting Bayes factor means and ranges. We had to deviate from the preregistered analysis plan and reduce the number of samples to 100, 000 and the number of repeated computations (two for the specified statistical model and one for each robustness tests) to make our extensive analyses computationally feasible. Running the analysis with fewer samples had no negative influence on chain convergence.

In study 1, we observed a bimodal distribution for the group-level mean and standard deviation of the vagueness parameter at time point 1. Running the analysis with more samples did not improve the convergence of the chain. However, we found that participant 44 caused this abnormal chain behavior. Despite a slight monotonically increasing pattern, their percentage of TRUE responses for this participant never exceeded 40%, which is atypical compared to the other test subjects and suggests that they did not properly adhere to task instructions. We deviated from our preregistered plan and excluded this participant’s responses for the quantifier *more than half* at time points 1 and 2. This exclusion substantially improved chain convergence for the vagueness parameter at time point 1 and did not substantially alter estimates at time point 2 or the results of our hypothesis tests. Additionally, we conducted a robustness analysis using a high $\rho$ preferring prior on the correlation parameters $\rho _\beta$ and $\rho _\gamma$.

For study 2 the initial number of recruited participants (i.e., 248) was lower than the preregistered target of 300 since we exhausted our resources before reaching the target sample size. For both studies we deviated from the preregistration with respect to the data preprocessing. We initially proposed to transform the response data for the quantifier *many* such that the theoretically assumed vagueness above the meaning threshold would instead appear below the threshold. This transformation, however, changes the interpretation of the model parameters for this quantifier which would be at odds with the conceptual structure of the model and make it unsuitable for model comparison (e.g., the meaning threshold for *many* would then refer to the point after which all individuals consider the quantifier TRUE instead of representing the necessary and sufficient condition for the truth of the quantifier). We therefore deviated from the preregistered model and did not transform the response data for this quantifier. Additionally, we adjusted the transformation of response data for the quantifiers *few* and *fewer than half* (i.e., instead of flipping TRUE and FALSE responses as described in the preregistration, we reversed the presented percentages), as the original transformation was incorrect and would have altered the interpretation of the model parameters as well. The deviations in data preprocessing did not substantially affect the results for our hypothesis test.

Finally, we corrected a coding error related to hypothesis $\mathcal {H}_d$. While we preregistered that we expected more response noise and a higher threshold for *most* compared to *more than half*, the initial code incorrectly tested whether *most* had less response noise and a lower threshold. Readers can access the results of our preregistered analysis, that is, the analysis before adjusting the data preprocessing and before excluding participant 44 in study 1 in our online supplements at: https://doi.org/10.17605/OSF.IO/NY73W.

## Figures and Tables

**Fig. 11:**
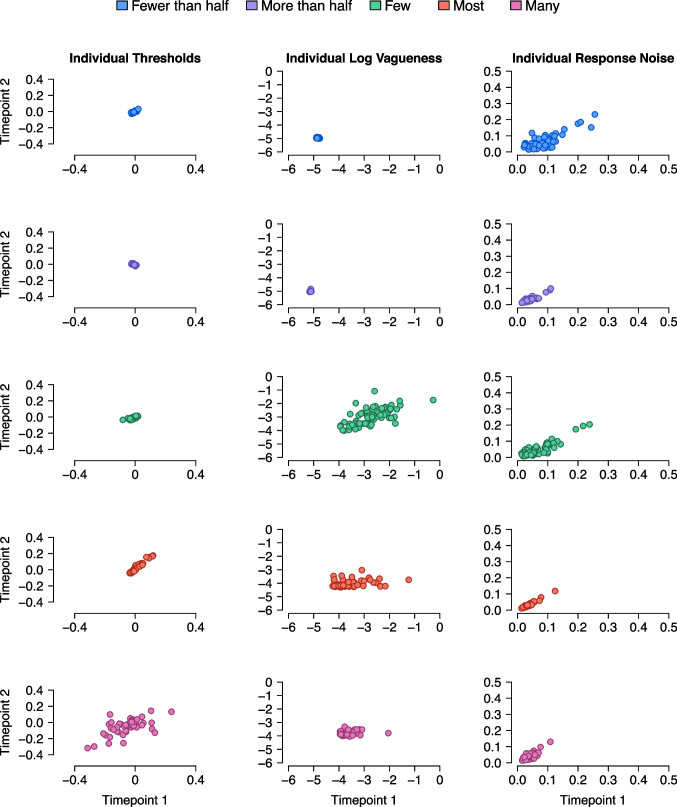
Relationships between the posterior medians of individual-level thresholds, log-vagueness, and response noise at time point 1 and time point 2 in study 1

**Fig. 13:**
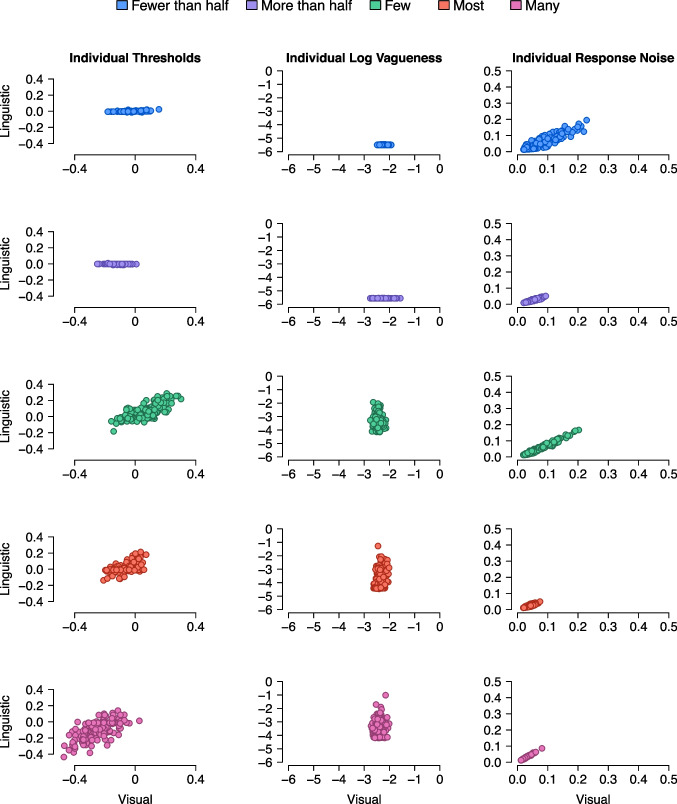
Relationships between the posterior medians of individual-level thresholds, log-vagueness, and response noise across the visual and the linguistic paradigm

## Data Availability

Readers can access the preregistration, data, code to reproduce our materials, and the R code to conduct all analyses (including all figures) in our OSF folder at https://osf.io/ny73w/ (study 1) and https://osf.io/tygbf/  (study 2).

## Appendix A: Report of Remaining Preregistered Hypotheses

In addition to the predictions outlined in the main text, we preregistered additional hypotheses, the results of which we present below. These hypotheses examine the ordering of cognitive parameters observed in Ramotowska et al. (2023) and Ramotowska et al. (2024), test whether vagueness exists at both the group and individual levels, and examine whether differences between *most* and *more than half* can be detected in the visual and linguistic paradigm.

### Study 1

The following additional research questions and hypotheses were preregistered for study 1:

- Are there differences in vagueness on the group and individual levels?
  $\mathcal {H}_{a,1}$: We build on the findings that *most* exhibits greater vagueness compared to *more than half* and generalize this prediction to quantifiers with sharp meaning thresholds compared to those with vague meaning thresholds. That is, we predict that at the group level, the order of the vagueness parameter is (from least vague to most vague): (*more than half*, *fewer than half*) < (*most*, *many*, *few*). The alternative hypothesis allows all parameters to vary freely.
  $\mathcal {H}_{a,2}$: A model that estimates individual-level vagueness outperforms an alternative model that assumes a fixed and very low vagueness for each individual.
- Can we determine an order of response noise rate for quantifiers, based on the ordering of non-decision times found in Ramotowska et al. (2023)?
  $\mathcal {H}_{b,1}$: On the group level, the order of response noise rate (from least noise rate to most noise rate) is: *more than half* < ( *many*, *most*) < *fewer than half* < *few*, corresponding to an interaction between the vagueness of the meaning threshold and its entailment (i.e., whether the quantifier is upward-entailing or downward-entailing). The alternative hypothesis allows all parameters to vary freely.
  $\mathcal {H}_{b,2}$: As a secondary analysis, we will test the main effects that follow from the interaction specified in $\mathcal {H}_{b,1}$. First we tested whether, on the group level, the order of response noise rate (from least error rate to most error rate) is associated with whether the quantifier is upward-entailing or downward-entailing: ( *more than half*, *many*, *most*) < ( *fewer than half*, *few*). The alternative hypothesis allows all parameters to vary freely.
  $\mathcal {H}_{b,3}$: Second we tested whether, on the group level, the order of response noise rate (from least error rate to most error rate) is associated with the vagueness of the quantifier: ( *more than half*, *fewer than half*) < ( *many*, *most*, *few*). The alternative hypothesis allows all parameters to vary freely.

The log Bayes factors relating for each additional preregistered hypothesis are summarized in Table 3. The first research question related to the vagueness around the meaning threshold and the question of whether *most* could be classified as a vague quantifier. We found strong evidence in favor of the hypothesis that quantifiers with vague meaning thresholds (i.e., *few*, *many*, *most*) exhibit greater vagueness compared to quantifiers with sharp meaning thresholds (i.e., *fewer than half*, *more than half*); $\text {BF}_{12} = \text {exp}(\text {logBF}_{12}) = 98$. Furthermore, the findings of this study indicate that the meaning of quantifiers can be most effectively captured by a computational model that incorporates individual-level vagueness. Specifically, we found overwhelming evidence in favor of a model that estimates individual-level vagueness compared to an alternative model that assumes a fixed and very low vagueness for each individual; $\text {BF}_{12} = \text {exp}(\text {logBF}_{12}) > 1 \times 10^{304}$. As an additional exploratory analysis, we investigated whether this result holds for each quantifier. Ramotowska et al. (2023) found that for the quantifiers *more than half* and *fewer than half* a model without vagueness fit the data better for the majority of participants. Our analyses did not confirm this finding; we found overwhelming evidence in favor of a model that estimates individual-level vagueness for each quantifier, including *more than half*, $\text {BF}_{12} = 7.13 \times 10^{30}$, and *fewer than half*, $\text {BF}_{12} = 3.96 \times 10^{46}$. These results were stable over repeated computations and different specifications of parameter priors.

**Table 3:** Log Bayes factors in favor of the hypothesis of interest ($\mathcal {H}_1$) compared to the alternative hypothesis ($\mathcal {H}_2$) for study 1. See the main text for explanations regarding the competing hypotheses

	Main Analysis		Robustness Checks		
Hypothesis of Interest	Rep. 1	Rep. 2	Narrow Prior	Wide Prior	High $\rho$ Prior
$\mathcal {H}_{a,1}$	4.58	4.67	4.58	4.54	4.61
$\mathcal {H}_{a,2}$	789.34	819.80	814.83	780.38	840.94
$\mathcal {H}_{b,1}$	3.91	3.81	3.68	4.29	3.83
$\mathcal {H}_{b,2}$	4.56	4.55	4.67	4.38	4.54
$\mathcal {H}_{b,3}$	$<-6.93$	$<-6.89$	$<-6.86$	$<-6.92$	$<-6.98$

*Note.* A log Bayes factor higher than 2.3 indicates strong evidence ($\text {BF}_{12}>$ 10) in favor of the hypothesis of interest. A log Bayes factor higher than 4.6 indicates overwhelming evidence ($\text {BF}_{12}>$ 100) in favor of the hypothesis of interest. Negative log Bayes factors indicate evidence in favor of the alternative hypothesis. A ’<’ sign in a Bayes factor indicates that we reached the maximum resolution with the given number of prior and posterior samples

The second research question related to the order of response noise of quantifiers for both time points. For this research question, we confirmed our prediction that upward-entailing quantifiers exhibited less response noise than downward-entailing quantifiers and that for a given entailment, quantifiers with sharp meaning thresholds exhibit less response noise than quantifiers with vague meaning thresholds. That is, we received strong evidence, $\text {BF}_{12} = \text {exp}(\text {logBF}_{12}) = 50$, in favor of the hypothesis that response noise is ordered as follows: *more than half* < (*many, most*) < *fewer than half* < *few*. The secondary analyses of this hypothesis examined the two main effects of the interaction separately: the main effect of quantifier entailment and the main effect of the vagueness of the meaning threshold. We received strong evidence that upward-entailing quantifiers are associated with lower response noise than downward-entailing quantifiers; $\text {BF}_{12} = \text {exp}(\text {logBF}_{12}) = 96$. However, we did not find support in favor of the main effect of the vagueness of meaning thresholds. When quantifier entailment was disregarded, we received overwhelming evidence *against* the hypothesis that individuals exhibit less response noise for quantifiers with a sharp meaning threshold compared to quantifiers with a vague meaning threshold; $\text {BF}_{21} = 1/\text {exp}(\text {logBF}_{12}) \approx 1022$. The results of the these analyses are consistent over repeated computations. In addition, for all hypotheses, we yield qualitatively identical results over our robustness conditions with different prior distributions.

### Study 2

The following additional research questions and hypotheses were preregistered for study 2:

- Can we predict the ordering of vagueness and response noise in the visual paradigm?
  $\mathcal {H}_{c,1}$: At the group level, we predicted that in the visual paradigm the order of the vagueness parameter and the response noise parameter would align with the vagueness of the quantifiers’ meaning thresholds: (*more than half*, *fewer than half*) < (*most*, *many*, *few*). The alternative hypothesis allows all parameters to vary freely.
  $\mathcal {H}_{c,2}$: We then tested predictions of the ordering of the vagueness parameters and response noise separately, based on the results by Ramotowska et al. (2024). Regarding the vagueness parameters we predicted that, at the group level, the order of the vagueness parameter is (from least vague to most vague): (*more than half*, *fewer than half*) < *most* < ( *many*, *few*). The alternative hypothesis lets all parameters vary freely.
  $\mathcal {H}_{c,3}$: In addition, we tested the alternative ordering of response noise parameter based on the entailment of the quantifier. That is, at the group level, in the visual paradigm the order of response noise rate (from least error rate to most error rate) is: ( *more than half*, *many*, *most*) < ( *fewer than half*, *few*). The alternative hypothesis lets all parameters vary freely.
- Do we find differences between *most* and *more than half* in both tasks?
  $\mathcal {H}_{d}$: Building on the findings by Ramotowska et al. (2023) that *most* and *more than half* differ in vagueness and non-decision time within the linguistic paradigm, we generalize this expectation to the visual paradigm. We predict to find more response noise for *most* than *more than half*, and a higher threshold for *most* than *more than half* in both tasks. The alternative hypothesis predicts that in both tasks the two quantifiers are equivalent.

Results of the hypothesis tests are summarized in Table 4. Our first research question concerned the ordering of vagueness and response noise in the visual paradigm. We hypothesized that the order of vagueness and response noise parameters are determined by whether the corresponding quantifier had a sharp or vague meaning threshold. Specifically, we predicted that *more than half* and *fewer than half* would show lower vagueness and less response noise than *most*, *many*, and *few*. We found overwhelming evidence against this hypothesis, $\text {BF}_{21} = 1/\text {exp}(\text {logBF}_{12}) < 1054$. Vagueness was nearly identical for all quantifiers in the visual paradigm, and response noise was ordered based on the quantifier’s entailment and not based on whether it has a sharp or vague meaning threshold.

**Table 4:** Log Bayes factors in favor of the hypothesis of interest ($\mathcal {H}_1$) compared to the alternative hypothesis ($\mathcal {H}_2$) for study 2. See the main text for explanations regarding the competing hypotheses

	Main Analysis		Robustness Checks	
Hypothesis of Interest	Rep. 1	Rep. 2	Narrow Prior	Wide Prior
$\mathcal {H}_{c,1}$	$<-6.96$	$<-6.92$	$<-6.72$	$<-6.89$
$\mathcal {H}_{c,2}$	$<-8.18$	-6.54	$<-8.09$	$<-8.19$
$\mathcal {H}_{c,3}$	2.32	2.29	2.32	2.28
$\mathcal {H}_{d}$	-0.86	-0.76	-1.73	0.48

*Note.* A log Bayes factor higher than 2.3 indicates strong evidence ($\text {BF}_{12}>$ 10) in favor of the hypothesis of interest. A log Bayes factor higher than 4.6 indicates overwhelming evidence ($\text {BF}_{12}>$ 100) in favor of the hypothesis of interest. Negative log Bayes factors indicate evidence in favor of the alternative hypothesis. A ’<’ sign in a Bayes factor indicates that we reached the maximum resolution with the given number of prior and posterior samples

Furthermore, based on the results by Ramotowska et al. (2024), we hypothesized for the visual paradigm a more specific ordering of the vagueness parameters based on whether the quantifier has sharp or vague meaning thresholds: (*more than half*, *fewer than half*) < *most* < (*many*, *few*). Again, we found overwhelming evidence against this hypothesis, with $\text {BF}_{21} = 1/\text {exp}(\text {logBF}_{12}) < 3569$. Results related to the these research questions were robust against multiple repetitions and alternative plausible prior specifications. Lastly, we hypothesized that for the visual paradigm the order of response noise would be based on the quantifier’s entailment, which too was based on results found in a linguistic paradigm by Ramotowska et al. (2024). We found strong evidence supporting this hypothesis, with $\text {BF}_{12} = \text {exp}(\text {logBF}_{12}) = 10.2$. For this hypothesis, repeated computations and alternative prior specifications just missed our preregistered evidence threshold of 10. The Bayes factor of the second repetition of the main analysis and the wide prior suggests moderate evidence in favor for our hypothesis, with $\text {BF}_{12} = \text {exp}(\text {logBF}_{12}) = 9.9$ and $\text {BF}_{12} = \text {exp}(\text {logBF}_{12}) = 9.8$, respectively.

Building on the findings of Ramotowska et al. (2023), our last research question addressed potential differences between *most* and *more than half* in both paradigms. We hypothesized that *most* would be associated with higher response noise than *more than half* and that *most* would have a higher meaning threshold than *more than half*. The data was inconclusive for this hypothesis. We found anecdotal evidence *against* this prediction, with $\text {BF}_{21} = 1/\text {exp}(\text {logBF}_{12}) = 2.36$ in favor for the null hypothesis that both quantifiers are equal. This result was not stable against alternative prior specifications; the Bayes factor related to the wide prior suggested inconclusive evidence, with $\text {BF}_{12} = \text {exp}(\text {logBF}_{12}) = 1.62$.

## Appendix B: Methodologies to Compute the Bayes Factor

We answered our research questions and tested our hypotheses by means of Bayes factors (Jeffreys, 1935; Kass & Raftery, 1995), the standard way to test hypotheses within the Bayesian framework. The Bayes factor is defined as the ratio of marginal likelihoods of two competing models. It indicates the extent to which the data support one model over the other and is given by:

C1 $$\begin{aligned} \text {BF}_{12} = \frac{\overbrace{p(\text {data}\mid \mathcal {H}_1)}^{\begin{array}{c} \text {Marginal likelihood}\\ \text {under}~\mathcal {H}_1 \end{array}}}{\underbrace{p(\text {data}\mid \mathcal {H}_2)}_{\begin{array}{c} \text {Marginal likelihood}\\ \text {under}~\mathcal {H}_2 \end{array}}}. \end{aligned}$$

Computing the marginal likelihoods requires integrating over the entire parameter space, which is particularly challenging for complex models like the one proposed here. As a solution, researchers resort to Markov-Carlo sampling methods to perform the desired comparisons and have derived Bayes factor identities to facilitate the calculations. Here, we use three different methods to approximate Bayes factors, which we use to test our hypotheses. The first is the bridge sampling method (Gronau et al., 2020, 2017; Meng & Wong, 1996), which can be used when comparing two hypotheses that can be implemented as computational models. The method is then applied to each computational model separately and yields an estimate of the marginal likelihoods. In study 1, we employ this method to test the stability of semantic representation across time (hypothesis 1.1, study 1) and the absence of vagueness (hypothesis a.2, study 1) as we implemented these hypotheses as competing computational models.

Informed hypotheses, such as expected order among parameters, are not easily implemented in the computational model itself and thus require a different methodology. To test an encompassing (or unconstrained) hypothesis $\mathcal {H}_e$, which permits any order among parameters, against the informed hypothesis $\mathcal {H}_r$, we used the unconditional encompassing method (Klugkist et al., 2005). The Bayes factor $\text {BF}_{er}$, where the subscripts *e* and *r* refer to $\mathcal {H}_e$ and $\mathcal {H}_r$, respectively, is defined as the ratio of proportions of the posterior and prior parameter space in agreement with the restriction:

C2 $$\begin{aligned} \text {BF}_{er} = \frac{\overbrace{p(\boldsymbol{\theta } \in \mathcal {R}(\theta ) \mid \mathcal {H}_e)}^{\begin{array}{c} \text {Proportion of prior parameter}\\ \text {space consistent with the restriction} \end{array}}}{\underbrace{p(\boldsymbol{\theta } \in \mathcal {R}(\theta ) \mid \text {data}, \mathcal {H}_e)}_{\begin{array}{c} \text {Proportion of posterior parameter}\\ \text {space consistent with the restriction} \end{array}}}, \end{aligned}$$

where $\mathcal {R}(\theta )$ denotes the restricted parameter space of $\boldsymbol{\theta }$. Using this identity, the approximation of $\text {BF}_{er}$ is simple. It requires only samples from the posterior and prior distribution of the encompassing hypothesis. Then, it is possible to estimate $p(\boldsymbol{\theta } \in \mathcal {R}(\theta ) \mid \mathcal {H}_e)$ and $p(\boldsymbol{\theta } \in \mathcal {R}(\theta ) \mid \text {data}, \mathcal {H}_e)$ through the sample mean of the prior and posterior samples that obey the constraint. The unconditional encompassing method was employed to test most hypotheses in this manuscript, specifically hypotheses a.1, b.1, b.2, b.3 in study 1, as well as all hypotheses in study 2.

A special case of the unconditional encompassing approach is the so-called Savage-Dickey density ratio (Gelfand et al., 1992; Sedransk et al., 1985; Klugkist et al., 2005). The Savage-Dickey density ratio is mainly used to test point estimates of parameters or test whether two parameters are exactly equal (i.e., their difference is zero), denoted as $\mathcal {H}_0$. Approximating the parameter space in accordance with the restriction for the point null hypothesis is not possible, as it is, by definition, zero. Instead, it was proposed to divide the height of the posterior distribution at the point of interest (i.e., $\textbf{c}$) by the height of the prior distribution at the same point $\text {BF}_{0e}$ (Dickey & Lientz, 1970; Dickey, 1971; O’Hagan & Forster, 2004; Verdinelli & Wasserman, 1995). The Bayes factor $\text {BF}_{0e}$ is then given by:

C3 $$\begin{aligned} \text {BF}_{0e} = \frac{p(\textbf{x} \mid \mathcal {H}_0)}{p(\textbf{x} \mid \mathcal {H}_e)} = \frac{\overbrace{p(\boldsymbol{\theta } = \textbf{c} \mid \textbf{x}, \mathcal {H}_e)}^{\begin{array}{c} \text {Height of posterior density of}~\mathcal {H}_e\\ \text {at}~\theta = c \end{array}}}{\underbrace{p(\boldsymbol{\theta } = \textbf{c} \mid \mathcal {H}_e)}_{\begin{array}{c} \text {Height of prior density of}~\mathcal {H}_e\\ \text {at}~\theta = c \end{array}}}. \end{aligned}$$

Due to the absence of a closed-form solution for the posterior distributions in our model and the potential vulnerability of our model to opaque priors (Merkle et al., 2023), we employed logspline nonparametric density estimates to approximate the prior and posterior densities and compute the height of the densities at the point of interest (Kooperberg, 2020; Stone et al., 1997). The hypotheses of interest 2.1, 2.2, and hypothesis d in study 2 predict an ordinal pattern, while the alternative hypotheses predict equality of parameters. To compare the informed hypotheses against the exact-equality constrained hypotheses, we first computed the Bayes factor $\text {BF}_{er}$ using the unconditional encompassing method, then computer the Bayes factor $\text {BF}_{0e}$ using the Savage-Dickey density ration, and finally computing $\text {BF}_{r0}$ by multiplying both quantities.

## Appendix C: Parameter Estimates

### Study 1

The response pattern in study 1 highlights differences in meaning between the quantifiers and slight differences between time points. The correlation between time points for the meaning thresholds were high. The highest correlation between thresholds is estimated for the quantifier *most* (0.92[0.77, 0.98]), followed by *many* (0.55[0.31, 0.73]). The correlations for the quantifiers *more than half*, *fewer than half*, and *few* could not be reliably estimated supposedly due to limited variability in thresholds between participants, a challenge also noted in the original study (see also Rouder et al. (2023) for an extensive discussion on the difficulties of with measuring correlations with small degrees of individual variability in cognitive tasks). For these quantifiers, the credible intervals for the estimates nearly span the entire scale: *fewer than half* had a median posterior estimate of 0.88 with a 95% credible interval ranging from $-0.77$ to 0.97; *few* had a median estimate of 0.74 with a 95% credible interval ranging from $-0.63$ to 0.95; *more than half* was estimated with even greater uncertainty, with a median posterior estimate of $-0.92$ and a 95% credible interval ranging from $-0.98$ to 0.96.

Regarding the correlation between response noise parameters, upward-entailing quantifiers with a sharp meaning threshold were associated with higher correlations (*more than half*: 0.83[0.39, 0.97], *most*: 0.91[0.65, 0.98]), compared to the remaining quantifiers (*fewer than half*: 0.67[0.36, 0.87], few: 0.79[0.55, 0.92], many: 0.68[0.05, 0.93]). Figure 11 displays the relationships between the posterior medians of individual-level thresholds, log-vagueness, and response noise at time point 1 and time point 2.

The group-level estimates for the meaning threshold (on its original scale), vagueness, and response noise are depicted in Fig. 5. To compare results from our proposed model with standard probabilistic models of meaning, we will present both the meaning threshold (the necessary and sufficient condition for a quantifier to be TRUE) and the point where the probability of considering the quantifier as TRUE reaches 50%. The meaning thresholds for *more than half* are $49\,[49, 50]$ for time point 1 and $49\,[48, 50]$ for time point 2. Almost identical, for *fewer than half* they are $51\,[50, 52]$ for time point 1 and $50\,[50, 51]$ for time point 2. The probability of responding TRUE for both quantifiers at time point 1 and time point 2 reaches 50% at $50\%$.

For the quantifier *most*, the meaning threshold is slightly higher than for *more than half*, that is, $50\,[49, 52]$ at time point 1 and increasing slightly to $51\,[49, 53]$ at time point 2. The $50\%$ probability of answering TRUE for this quantifier is reached at $52\%$ for both time points. The meaning threshold for the quantifier *many* falls slightly below $50\%$, specifically $45\,[43,48]$ at time point 1 and $46\,[43,49]$ at time point 2. The $50\%$ probability of responding TRUE for this quantifier occurs at $47\%$ for both time points. In contrast, the meaning threshold for *few* is estimated at $52\,[51,53]\%$ for time point 1 and $51\,[50,52]$ for time point 2. The $50\%$ probability of responding TRUE for this quantifier occurs at $48\%$ at both time points.

The quantifiers *more than half* and *fewer than half* exhibit highly similar and low levels of vagueness. For time point 1, the log vagueness of *more than half* is $-5.13\,[-5.57, -4.59]$, and for *fewer than half*, it is $-4.83\,[-5.43, -4.09]$. For time point 2, both quantifiers show even greater similarity: the log vagueness of *more than half* is $-4.99\,[-5.52, -4.44]$, and for *fewer than half*, it is $-4.95\,[-5.49, -4.37]$.

Interestingly, the difference in vagueness between *most* and *many* increases at time point 2. The vagueness for *most* decreases from $-3.64\,[-4.04, -3.27]$ to $-4.07\,[-4.54, -3.67]$, while the vagueness for *many* remains relatively stable around $-3.70$ (i.e., $-3.68\,[-4.12, -3.31]$ at time point 1 and $-3.82\,[-4.45, -3.37]$ at time point 2). The quantifier *few* is associated with the highest uncertainty around the meaning threshold, with a vagueness parameter of $-2.78\,[-3.11, -2.50]$ at time point 1 and $-2.99\,[-3.30, -2.73]$ at time point 2.

In terms of response noise, the model estimates suggest a clear distinction between upward-entailing and downward-entailing quantifiers. Upward-entailing quantifiers show low response noise parameters at both time points, ranging between 0.04 and 0.03. At time point 1, downward-entailing quantifiers exhibit slightly higher response noise, specifically $0.09\,[0.07, 0.11]$ for *fewer than half* and $0.08\,[0.06, 0.09]$ for *few*. By time point 2, this noise decreases to $0.07\,[0.05, 0.08]$ for *fewer than half* and $0.06\,[0.04, 0.07]$ for *few*.

**Table 5:** Median and 95 % credible intervals of group-level model parameters for each quantifier for study 1. The subscripts indicate whether the estimate refers to time point 1 or time point 2. Note that the threshold parameters for the quantifiers *fewer than half*, *few*, and *many* were estimated by flipping data; negative values for these parameters refer to thresholds above 50 in the original scale

Parameter	Fewer than half	More than half	Few	Most	Many
$\mu _{\text {log}(\alpha , 1)}$	-4.83 [-5.43, -4.09]	-5.13 [-5.57, -4.59]	-2.78 [-3.11, -2.50]	-3.64 [-4.04, -3.27]	-3.68 [-4.12, -3.31]
$\mu _{\text {log}(\alpha , 2)}$	-4.95 [-5.49, -4.37]	-4.99 [-5.52, -4.44]	-2.99 [-3.30, -2.73]	-4.07 [-4.54, -3.67]	-3.82 [-4.45, -3.37]
$\mu _{\beta , 1}$	-0.01 [-0.02, 0.00]	-0.01 [-0.01, 0.00]	-0.02 [-0.03, -0.01]	0.00 [-0.01, 0.02]	-0.05 [-0.07, -0.02]
$\mu _{\beta , 2}$	0.00 [-0.01, 0.00]	-0.01 [-0.02, 0.00]	-0.01 [-0.02, 0.00]	0.01 [-0.01, 0.03]	-0.04 [-0.07, -0.01]
$\mu _{\gamma , 1}$	0.09 [0.07, 0.11]	0.04 [0.03, 0.05]	0.08 [0.06, 0.09]	0.03 [0.02, 0.05]	0.04 [0.03, 0.05]
$\mu _{\gamma , 2}$	0.07 [0.05, 0.08]	0.03 [0.02, 0.04]	0.06 [0.04, 0.07]	0.03 [0.02, 0.04]	0.04 [0.03, 0.05]
$\sigma _{\text {log}(\alpha , 1)}$	0.31 [0.14, 0.91]	0.25 [0.13, 0.47]	0.85 [0.64, 1.14]	0.89 [0.64, 1.18]	0.66 [0.22, 1.03]
$\sigma _{\text {log}(\alpha , 2)}$	0.28 [0.14, 0.54]	0.29 [0.13, 0.55]	0.79 [0.58, 1.07]	0.60 [0.24, 0.92]	0.49 [0.19, 0.87]
$\sigma _{\beta , 1}$	0.03 [0.02, 0.04]	0.03 [0.02, 0.04]	0.04 [0.03, 0.05]	0.05 [0.04, 0.06]	0.10 [0.08, 0.13]
$\sigma _{\beta , 2}$	0.03 [0.02, 0.04]	0.03 [0.02, 0.04]	0.03 [0.02, 0.04]	0.07 [0.05, 0.08]	0.10 [0.08, 0.12]
$\sigma _{\gamma , 1}$	0.06 [0.05, 0.08]	0.04 [0.03, 0.05]	0.05 [0.04, 0.07]	0.03 [0.03, 0.04]	0.03 [0.03, 0.04]
$\sigma _{\gamma , 2}$	0.05 [0.04, 0.07]	0.03 [0.02, 0.04]	0.05 [0.04, 0.06]	0.03 [0.02, 0.04]	0.03 [0.03, 0.05]
$\rho _\beta$	0.88 [-0.77, 0.97]	-0.92 [-0.98, 0.96]	0.74 [-0.63, 0.95]	0.92 [0.77, 0.98]	0.55 [0.31, 0.73]
$\rho _\gamma$	0.67 [0.36, 0.87]	0.83 [0.39, 0.97]	0.79 [0.55, 0.92]	0.91 [0.65, 0.98]	0.68 [0.05, 0.93]
$p(``TRUE'')_1 = 50\%$	50.05	49.75	47.65	52.25	47.15
$p(``TRUE'')_2 = 50\%$	49.95	49.85	48.05	52.35	47.25

### Study 2

The response pattern in study 2 highlights differences in meaning among the quantifiers and major differences between the tasks. Nevertheless, the correlation between thresholds in both tasks remains moderate to high for most quantifiers. The highest correlation between thresholds were found for the quantifier *few* with $0.69\,[0.58, 0.77]$, followed by *many* ($0.63\,[0.51, 0.72]$), and *most* ($0.51\,[0.36, 0.63]$). The correlation between thresholds for the quantifiers *fewer than half* ($0.34\,[-0.12, 0.69]$) and *more than half* ($-0.13\,[-0.59, 0.45]$) are low and include negative values. This could be due to the significant reduction in individual variability in the linguistic paradigm, particularly for the quantifier *more than half*, where variations in thresholds between participants almost disappear. The estimation difficulties for these quantifiers echo the ones we found in study 1.

The correlations between response noise rates were similarly high for all quantifiers. However, unlike study 1, we did not find that upward-entailing quantifiers were associated with higher correlations than downward-entailing quantifiers. The highest correlation were found between response noise across tasks for the quantifier *many* ($0.94\,[0.73, 0.99]$), followed by *few* ($0.90\,[0.72, 0.98]$), *more than half* ($0.84\,[0.47, 0.97]$), *fewer than half* ($0.75\,[0.54, 0.90]$), and finally, with *most* ($0.73\,[-0.12, 0.96]$) which featured a 95% credible interval that included negative correlations. Figure 13 shows the relationships between the posterior medians of individual-level thresholds, log-vagueness, and response noise across the visual and linguistic paradigms.

The exact estimates group-level estimates for the meaning threshold are presented in Table 6. Here again, when reporting the meaning threshold of quantifiers, we present both the meaning threshold and the point at which the probability of considering a quantifier statement to be TRUE is 50%.

We found substantial discrepancies between meaning thresholds in the visual and linguistic paradigm. Upward-entailing quantifiers *more than half*, *most*, and *many* but also for the downward-entailing quantifier *fewer than half* individuals on the group level adopt a lower threshold in the visual paradigm. The meaning threshold for the quantifier *more than half* is estimated to be $50\,[50, 50]$ in the linguistic paradigm and decreases to $39\,[38, 40]$ in the visual paradigm; it reaches the 50% probability of answering TRUE for *more than half* at $50\%$ in the linguistic paradigm and at $46\%$ in the visual paradigm. For *most*, the meaning threshold is estimated to be $51\,[50,52]$ in the linguistic paradigm and $42\,[41,44]$ in the visual paradigm; the 50% probability of answering TRUE for *most* is reached at $53\%$ in the linguistic paradigm and at $49\%$ in the visual paradigm. Similarly, for the quantifier *many*, the meaning threshold is estimated to be $40\,[39, 42]$ in the linguistic paradigm and $26\,[24,27]$ in the visual paradigm; the 50% probability for responding TRUE for this quantifier is reached at $43\%$ in the linguistic paradigm and at $32\%$ in the visual paradigm. Finally, we find the same trend for *fewer than half*. That is, the meaning threshold for this quantifier decreases from $50\,[50,50]$ in the linguistic paradigm to $48\,[47,50]$ in the visual paradigm. The 50% probability of responding TRUE is reached at $50\%$ in the linguistic paradigm and at $44\%$ in the visual paradigm. For the quantifier *few* individuals, at the group level, adopted a higher threshold in the visual paradigm. The meaning threshold for this quantifier rises from $56\,[55,58]$ in the linguistic paradigm to $57\,[55,59]$ in the visual paradigm. The point at which the quantifier reaches a $50\%$ probability of responding TRUE, however, shows the same pattern as the remaining quantifiers; in the linguistic paradigm, this point is reached at $41\%$, and in the visual paradigm, it is reached at $37\%$.

The differences in meaning thresholds between the visual and linguistic paradigms are also reflected in considerable differences in vagueness. As expected, we found higher vagueness estimates for all quantifiers in the visual paradigm compared to the linguistic paradigm. Interestingly, the vagueness estimates in the visual paradigm are nearly identical across all quantifiers, ranging from $-2.45\,[-2.56, -2.34]$ (*few*) to $-2.20\,[-2.32, -2.08]$ (*fewer than half*). For the linguistic paradigm, the vagueness estimates cluster together for quantifiers with vague and sharp meaning thresholds. The vagueness parameters for the quantifiers *fewer than half* and *more than half* are estimated to be $-5.49\,[-5.69, -5.23]$ and $-5.55\,[-5.74,-5.25]$, respectively. For *most*, vagueness is estimated to be $-3.76\,[-3.97,-3.56]$. The quantifiers *few* and *many* are almost indistinguishable with estimates of $-3.37\,[-3.57, -3.19]$ and $-3.37\,[-3.56, -3.20]$.

The ordering of response noise parameters in the linguistic paradigm aligns with the ordering we found in study 1. That is, we find lower response noise rates for upward-entailing quantifiers with estimates of $0.02\,[0.02,0.03]$ for *more than half* and *most*, and $0.03\,[0.03,0.04]$ for *many*. For the downward-entailing quantifiers, we received $0.06\,[0.06,0.07]$ for *fewer than half*, and $0.06\,[0.05,0.07]$ for *few*. The distinction between upward-entailing and downward-entailing quantifiers was found in the visual paradigm, as well. Among upward-entailing quantifiers, *many* had the lowest response noise estimate at $0.03\,[0.02, 0.04]$, followed by *most* ($0.04\,[0.03,0.05]$), and *more than half* ($0.05\,[0.04,0.06]$). For downward-entailing quantifiers, *few* is estimated to have lower response noise than *fewer than half*, with values of $0.08\,[0.07,0.09]$ and $0.09\,[0.08, 0.10]$, respectively.

**Table 6:** Median and 95 % credible intervals of group-level model parameters for each quantifier in study 2. The subscripts indicate whether the estimate refers to the visual paradigm (subscript 1) or the linguistic paradigm (subscript 2). Note that the threshold parameters for the quantifiers *fewer than half*, *few*, and *many* were estimated by flipping data; negative values for these parameters refer to thresholds above 50 in the original scale

Parameter	Fewer than half	More than half	Few	Most	Many
$\mu _{\text {log}(\alpha , 1)}$	-2.20 [-2.32, -2.08]	-2.32 [-2.42, -2.23]	-2.45 [-2.56, -2.34]	-2.35 [-2.45, -2.26]	-2.42 [-2.51, -2.33]
$\mu _{\text {log}(\alpha , 2)}$	-5.49 [-5.69, -5.23]	-5.55 [-5.74, -5.25]	-3.37 [-3.57, -3.19]	-3.76 [-3.97, -3.56]	-3.37 [-3.56, -3.20]
$\mu _{\beta , 1}$	-0.02 [-0.03, 0.00]	-0.11 [-0.12, -0.10]	0.07 [0.05, 0.09]	-0.08 [-0.09, -0.06]	-0.24 [-0.26, -0.23]
$\mu _{\beta , 2}$	0.00 [0.00, 0.00]	0.00 [0.00, 0.00]	0.06 [0.05, 0.08]	0.01 [0.00, 0.02]	-0.10 [-0.11, -0.08]
$\mu _{\gamma , 1}$	0.09 [0.08, 0.10]	0.05 [0.04, 0.06]	0.08 [0.07, 0.09]	0.04 [0.03, 0.05]	0.03 [0.02, 0.04]
$\mu _{\gamma , 2}$	0.06 [0.06, 0.07]	0.02 [0.02, 0.03]	0.06 [0.05, 0.07]	0.02 [0.02, 0.03]	0.03 [0.03, 0.04]
$\sigma _{\text {log}(\alpha , 1)}$	0.29 [0.17, 0.41]	0.36 [0.26, 0.45]	0.26 [0.15, 0.38]	0.24 [0.16, 0.32]	0.27 [0.18, 0.37]
$\sigma _{\text {log}(\alpha , 2)}$	0.16 [0.10, 0.26]	0.15 [0.09, 0.25]	0.72 [0.55, 0.90]	0.90 [0.74, 1.07]	0.78 [0.63, 0.94]
$\sigma _{\beta , 1}$	0.07 [0.06, 0.08]	0.05 [0.05, 0.06]	0.10 [0.09, 0.11]	0.07 [0.06, 0.08]	0.10 [0.09, 0.11]
$\sigma _{\beta , 2}$	0.01 [0.01, 0.01]	0.01 [0.01, 0.01]	0.09 [0.08, 0.10]	0.06 [0.05, 0.07]	0.11 [0.10, 0.13]
$\sigma _{\gamma , 1}$	0.06 [0.05, 0.07]	0.03 [0.02, 0.04]	0.05 [0.04, 0.06]	0.02 [0.02, 0.03]	0.02 [0.02, 0.03]
$\sigma _{\gamma , 2}$	0.04 [0.04, 0.05]	0.02 [0.02, 0.02]	0.04 [0.03, 0.05]	0.02 [0.01, 0.02]	0.02 [0.02, 0.03]
$\rho _\beta$	0.34 [-0.12, 0.69]	-0.13 [-0.59, 0.45]	0.69 [0.58, 0.77]	0.51 [0.36, 0.63]	0.63 [0.51, 0.72]
$\rho _\gamma$	0.75 [0.54, 0.90]	0.84 [0.47, 0.97]	0.90 [0.72, 0.98]	0.73 [-0.12, 0.96]	0.94 [0.73, 0.99]
$p(``TRUE'')_1 = 50\%$	43.94	45.65	36.84	49.15	31.93
$p(``TRUE'')_2 = 50\%$	49.85	50.05	41.44	52.55	42.74

## Footnotes

1 See the discussion of the relevant literature in Denic and Szymanik (2022).

2 A full-length New York City subway train can hold up to 1,500 passengers.

3 We use the term upward entailing to describe the following property: if a statement holds for a set, it also holds for any superset. For instance, if *more than half* of the dots are green in a display containing dots of different colors, then *more than half* of the dots are green or blue must also be true, since this property includes all green dots and possibly additional ones. In contrast, downward entailing quantifiers have the opposite property: if a statement holds for a set, it also holds for any subset. For instance, if *few* of the dots are green in a display containing dots of different shapes and sizes, then *few* of the dots are green and round must also be true, since this subset cannot contain more dots than the original set.

4 Initially, the data analysts aimed to set the cutoff criterion for slow responses at 7500ms. However, the data manager voiced concerns that the cutoff for slow criteria may be too lenient and based on inadequate visual representation of the response time distributions of the blinded data. Consequently, the visual representations were improved, and the response time distributions were reevaluated. Based on this reevaluation, the cutoff for slow responses was set to 5000ms.
